# Supplementary figures and images for: Antitumor effects of chloroquine/hydroxychloroquine mediated by inhibition of the NF-κB signaling pathway through abrogation of autophagic p47 degradation in adult T-cell leukemia/lymphoma cells
Source: PLoS One. 2021 Aug 18;16(8):e0256320. doi: 10.1371/journal.pone.0256320 (PMC8372904; doi:10.1371/journal.pone.0256320)

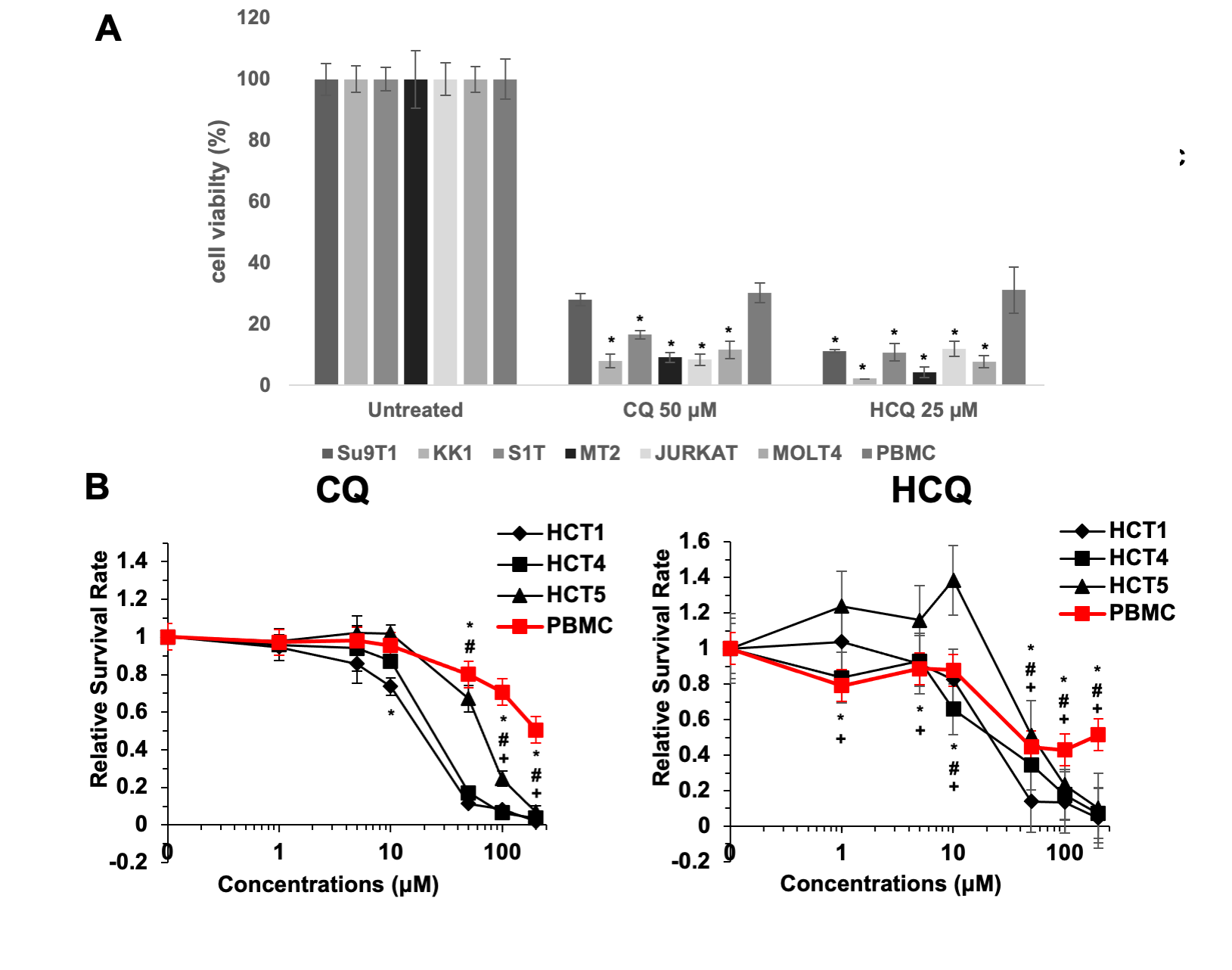

Supplement: S1 Fig — (A) Four ATLL cell lines (Su9T01, KK1, S1T, and MT2), two T-ALL cell lines (MOLT4 and JURKAT), and PBMCs from a healthy donor were treated with 50 μM CQ or 25 μM HCQ for 48 hours. Cell viability was determined by trypan blue. Relative cell viability was calculated based on the percentage of untreated cells (0 μM). Data are presented as the mean ± SD (n = 3). *p < 0.05 for Su9T01, KK1, S1T, MT2, MOLT4, or JURKAT cells compared to healthy PBMCs, respectively. (B) HCT1, HCT4, and HCT5 cell lines were treated with varying concentrations of CQ or HCQ for 48 hours. The relative cell viability was calculated as the percentage of untreated cells (0 μM). Data are presented as the mean ± SD (n = 3). *, #, or + P < 0.05, HCT1, HCT4, or HCT5 compared to Healthy PBMC, respectively. (TIF) [file pone.0256320.s001.tif]

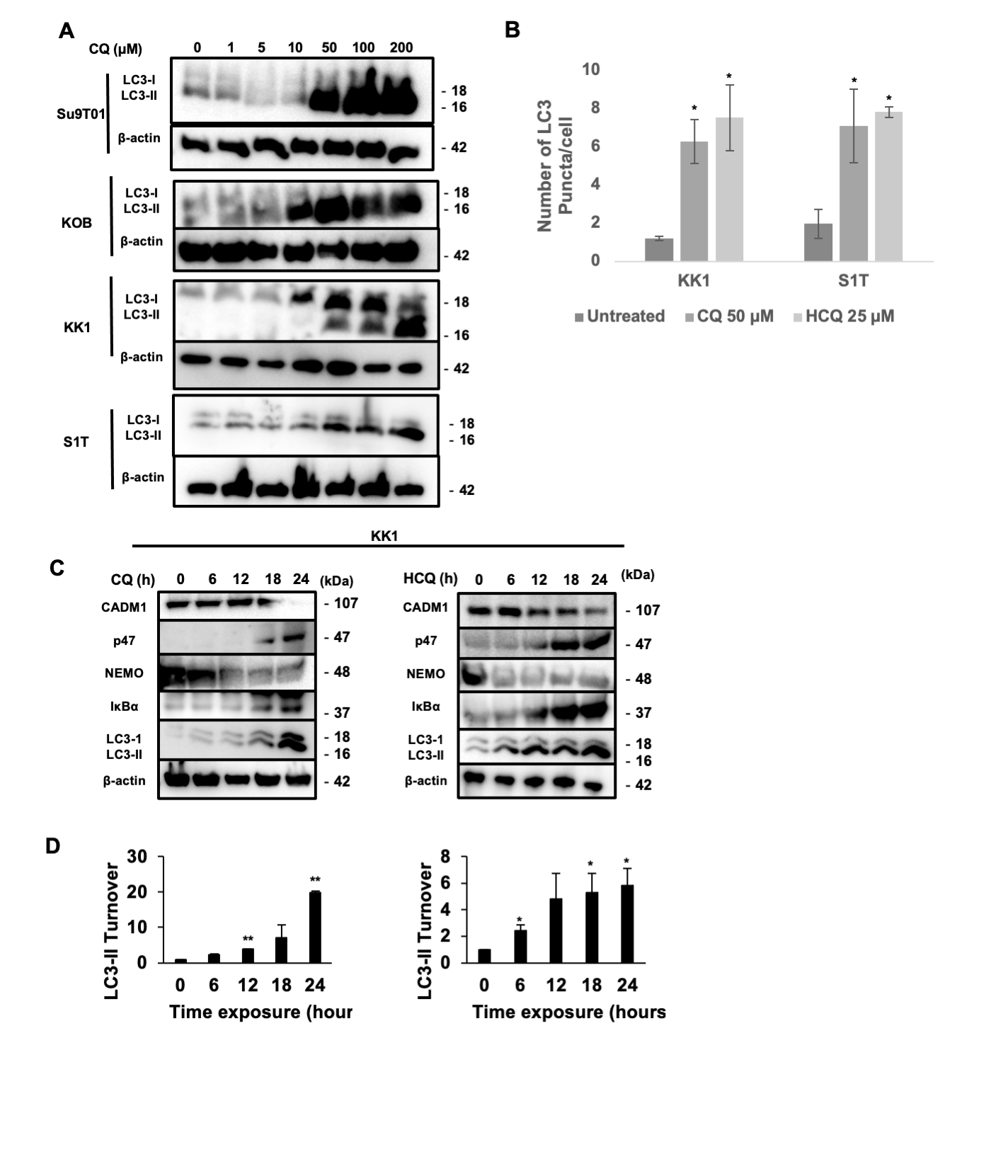

Supplement: S2 Fig — (A) Su9T01, KOB, KK1 and S1T cell lines were treated with varying concentrations of CQ for 24 hours. The expression of LC-3 was examined by Western blot analysis. (B) Quantification of LC3 puncta/cell is presented as the mean ± SD (n = 3, representative image was shown at Fig 2A). (C) Western blot analysis of CADM1, p47, and the indicated NF-κB and autophagy signaling proteins was performed in KK1 cell lines after treatment with either 50 μM CQ or 25 μM HCQ for 6, 12, 18, 24 hours. β-actin was used as a loading control. The cropped gels/blots are used in the figure, and the full-length gels/blots are presented in S1 Raw images. (D) LC3-II turnover are presented as the mean ± SD (n = 2 independent experiments, representative blot was shown at S2B Fig). *p < 0.05, **p<0.01 compared to control. (TIF) [file pone.0256320.s002.tif]

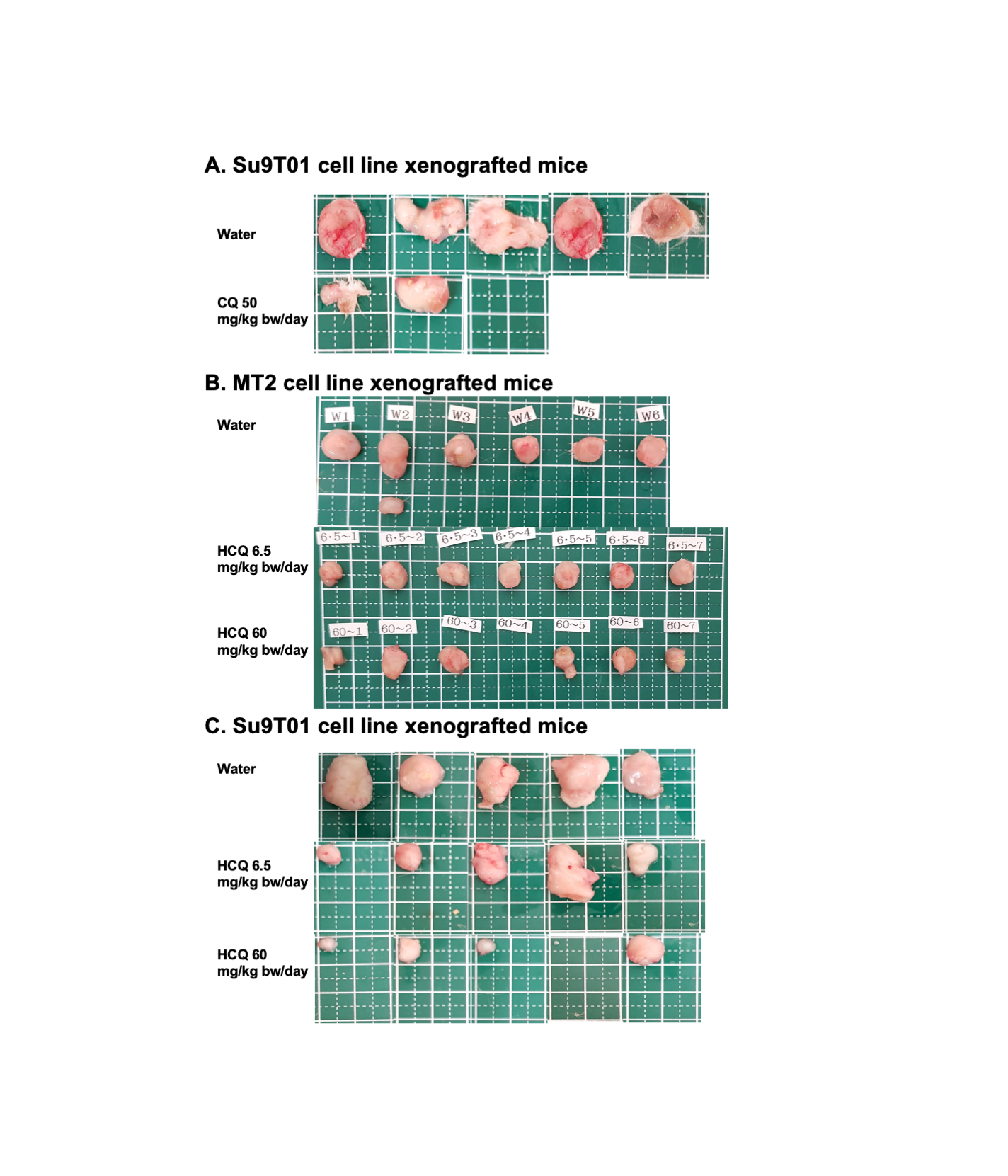

Supplement: S3 Fig — Photograph of a tumor isolated from mice bearing the indicated cell lines after treatment with CQ, HCQ, or water. (TIF) [file pone.0256320.s003.tif]

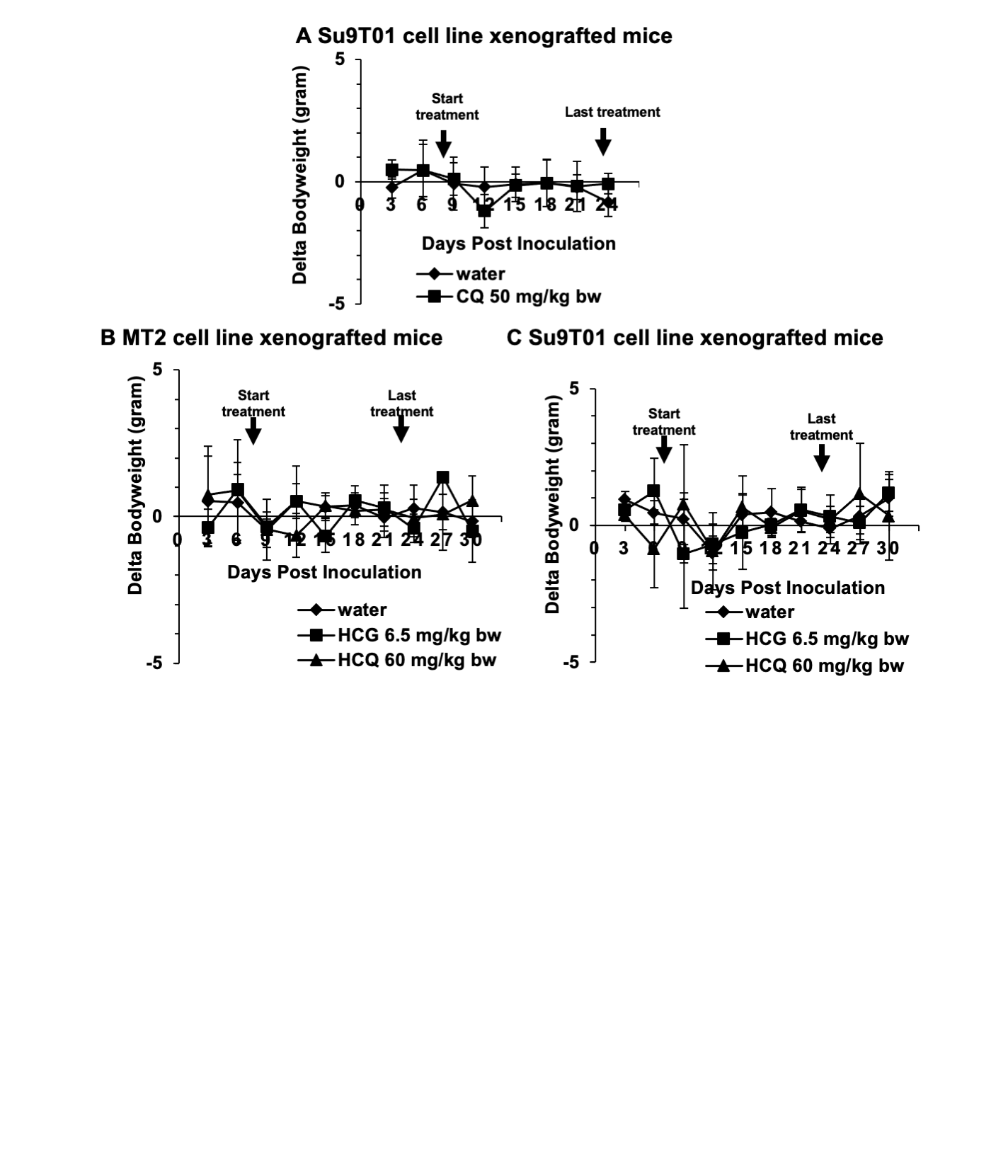

Supplement: S4 Fig — Records of weight variations of mice at every 3 days. Arrow indicates the time of treatment. (TIF) [file pone.0256320.s004.tif]
