# Supplementary material for: Antitumor effects of chloroquine/hydroxychloroquine mediated by inhibition of the NF-κB signaling pathway through abrogation of autophagic p47 degradation in adult T-cell leukemia/lymphoma cells
Source: PLoS One. 2021 Aug 18;16(8):e0256320. doi: 10.1371/journal.pone.0256320 (PMC8372904; doi:10.1371/journal.pone.0256320)
Supplement: S1 File — (PDF) [file pone.0256320.s005.pdf]

Supplementary Information for

Antitumor effects of chloroquine/hydroxychloroquine via inhibition of NF-κB signaling pathway through abrogation of p47 degradation by autophagy in adult T-cell leukemia/lymphoma cells

Yanuar Rahmat Fauzi, Shingo Nakahata, Syahrul Chilmi, Tomonaga Ichikawa, Phawut Nueangphuet, Ryoji Yamaguchi, Tatsufumi Nakamura Kazuya Shimoda, Kazuhiro Morishita

To whom correspondence should be addressed.

E-mail: kmorishi@med.miyazaki-u.ac.jp

Supplementary Tables 1-2

Supplementary Methods

Supplementary References

S1 Table. The IC50 value of CQ or HCQ in ATLL-derived and non-ATLL cell lines.

Cell line	Cell type	CQ (μM)	HCQ (μM)
Su9T01	ATLL-derived cell line	38.2	19.4
KK1	ATLL-derived cell line	44.1	44.2
S1T	ATLL-derived cell line	50.9	9.0
ST1	ATLL-derived cell line	26.9	30.8
HCT1	HAM-TSP	17.1	18.2
HCT4	HAM-TSP	23.9	29.8
HCT5	HAM-TSP	63.2	43.1
MOLT4	TALL	58.6	21.2
JURKAT	TALL	50.3	17.3

IC50, Concentration resulting in 50% inhibition of cell viability.

S2 Table. The IC50 value of CQ or HCQ in primary ATLL cells and PBMCs from a healthy donor.

Sample	Cell type	CQ (μM)	HCQ (μM)
Pt #1	Acute	21.7	13.5
Pt #2	Acute	18.5	16.2
Pt #3	Acute	18.3	10.5
PBMCs	Healthy donor	932.5	54.3

IC50, Concentration resulting in 50% inhibition of cell viability;

Supplementary Methods

Antibodies

The antibodies used for immunoblotting were Cleaved Caspase-3 (Asp175), IκBα (Mouse monoclonal antibody (mAb)), phospho-IκBα (Ser32/36) (5a5), phospho-eIF2α (Ser51), eIF2α (D7D3), and CHOP (L63F7) were from Cell Signaling Technology (Danvers, MA, USA). Anti-LC3B antibody was from Novus Biological (Centennial, Colorado, USA). Anti-NSFL1C and anti-β-actin (A441) antibodies were purchased from Sigma-Aldrich (Merck KGaA, Darmstadt, Germany). IKKγ/NEMO (FL-419) antibody was from Santa Cruz Biotechnology (Santa Cruz, CA, USA). The polyclonal antibody against human CADM1 was produced by immunizing rabbit with the human or humanC-terminal CADM1 peptide.

Reagents

CQ was purchased from R&D Systems (Minneapolis, USA). HCQ was from Sanofi under the trade name Plaquenil.

Western blot analysis

Cell samples were washed in ice-cold phosphate-buffered saline (PBS) twice and homogenized in boiling Sodium Dodecyl Sulfate (SDS) sample buffer (62.5 mM Tris-

HCl pH 6.8, 2% SDS, 25% Glycerol, 5% β-Mercaptoethanol, 0.01% Bromophenol blue). Protein samples were separated on 10% to 15% SDS-polyacrylamide gel and electroblotted onto Polyvinylidene Difluoride (PVDF) membrane (PVDF, Immobilon-P, Millipore, Billerica, MA). The membranes were blocked with 1% Bovine Serum Albumin (BSA, Nacalai Tesque, Kyoto, Japan) in Tris-buffered saline containing 0.1% Tween-20 (TBS-T) at room temperature for 1 h, then incubated at 4 °C overnight with primary antibody diluted in blocking buffer. They were then washed with TBS-T, incubated with corresponding horseradish peroxidase (HRP)-conjugated secondary antibodies in blocking buffer for 2 h at room temperature, and washed with TBS-T. The protein signal of western blot was visualized by chemiluminescence (EzWestLumi plus Western blotting reagents (6μl/cm^2), ATTO, Tokyo, Japan) and captured and quantified using either the LAS-3000 (Fujifilm, Tokyo, Japan) or Image Quant LAS-4000 (GE Healthcare Life Science, Tokyo, Japan).

Cell proliferation assay

Cells were cultured at 5 x 10^3 cells/well into the well of 96-well plate (Costar Corning, Rochester, NY) and treated with various concentrations of drugs in triplicate to a final volume of appropriate medium 100 μL. The plates were incubated at 37 °C in a humidified 5% CO_2 incubator for 48 h. After 48 h incubation, Cell proliferation assay

was performed using trypan blue exclusion method and Cell Counting Kit-8 (CCK-8; Dojindo Laboratories, Kumamoto, Japan). For CCK-8 method, 10 μL/well CCK-8 reagent solution was added, followed by incubation for 2 h in the same environment. Absorbance values were examined with a microplate reader (either ImmunoMini NJ-2300, Nalge Nunc International, Tokyo, Japan, or SpectraMax iD3 Multi-Mode Microplate Reader, Molecular Devices) at 450 nm. Values were normalized to control (water). IC50 was calculated as the drug concentration needed to reduce cell proliferation rate by 50%.

Apoptosis assay

Externalization of phosphatidylserine in apoptotic cells was evaluated by using the Annexin-V/DAPI double staining method. Briefly, cells were collected, washed with Annexin-V binding buffer twice, added 5 μl Annexin-V FITC (Biolegend) in 500 μl Annexin-V buffer, then incubated for 15 minutes in the dark at room temperature. Cells were washed once with Annexin-V binding buffer, added 1 μL DAPI (0.1 mg/ml), and then incubate for 5 min, and the samples were analyzed on a JSAN flow cytometer (Bay Bioscience, Kobe, Japan).

Hematoxylin eosin staining and immunohistochemistry

Samples were fixed in 10% buffered formalin solution (Mildform 20NM, Fujifilm, Wako, Japan), embedded in paraffin blocks, then 2-μm-thick sections were prepared. Slides were stained with hematoxylin and eosin (H&E) and IHC using the anti-cleaved caspase3 antibody (5A1E, Cell Signaling Technology, Danvers, MA, USA). The protocol has been described in detail elsewhere [1]. Quantification of anti-cleaved caspase-3 positive cells was performed by blinded manual counting the number of positive cells/High Power Field (HPF).

Immunofluorescence Microscopy

Cells were treated with 4% formaldehyde for 10 min at room temperature, washed once with 0.1 M glycine in TBS, and permeabilized with 0.1% Triton X-100 in TBS. After rewashing twice with 0.1 M glycine in TBS and blocking with 1% BSA in TBS, cells were incubated with anti-LC3 antibody from Novus Biology (1:200) overnight at 4 °C. The cells were then washed three times with TBS containing 0.1% Tween 20 and incubated with Alexa Fluor-488 anti-rabbit secondary antibody (Molecular Probes) at room temperature for 2 h. The coverslips were washed three times with TBS containing 0.1% Tween 20 and then mounted on glass slides using an antifade reagent (Invitrogen). Proteins were visualized using a confocal laser scanning microscope (Leica

Microsystems). Average numbers of LC3 puncta/cell were quantified by blinded manual counting of puncta, with at least 30 cells counted per group.

Statistical analysis

Data are shown as the mean ± standard deviation (s.d) from at least two independent repetitions. Western blot quantification was using ImageJ (NIH, USA). Basic assumption test, normality, and homogeneity test were performed to determine the statistical analysis, either parametric or non-parametric test. 2 group comparison was analyzed using either paired or independent two-tailed Student's t-test. Statistical analyses were performed using GraphPad Prism software (version 5.0, La Jolla, CA, USA), Microsoft Excel software program (Microsoft, WA, U.S.A.) and the Statistical Package for Social Sciences version 20.0 (SPSS Inc., Chicago, IL, USA). A p-value <0.05 was considered to have a statistically significant difference.

Supplementary References

1. Nakahata S, Ichikawa T, Maneesaay P, Saito Y, Nagai K, Tamura T, et al. Loss of NDRG2 expression activates PI3K-AKT signalling via PTEN phosphorylation in ATLL and other cancers. Nat Commun. Nature Publishing Group; 2014;5: 3393. doi:10.1038/ncomms4393
