# Supplementary material for: Antitumor effects of chloroquine/hydroxychloroquine mediated by inhibition of the NF-κB signaling pathway through abrogation of autophagic p47 degradation in adult T-cell leukemia/lymphoma cells
Source: PLoS One. 2021 Aug 18;16(8):e0256320. doi: 10.1371/journal.pone.0256320 (PMC8372904; doi:10.1371/journal.pone.0256320)
Supplement: S1 Raw images — (PDF) [file pone.0256320.s006.pdf]

Supplementary Information for

Antitumor effects of chloroquine/hydroxychloroquine via inhibition of NF-κB signaling pathway through abrogation of p47 degradation by autophagy in adult T-cell leukemia/lymphoma cells

Yanuar Rahmat Fauzi, Shingo Nakahata, Syahrul Chilmi, Tomonaga Ichikawa, Phawut Nueangphuet, Ryoji Yamaguchi, Tatsufumi Nakamura Kazuya Shimoda, Kazuhiro Morishita

To whom correspondence should be addressed. E-mail: kmorishi@med.miyazaki-u.ac.jp

Fig 1C

S1T-CQ

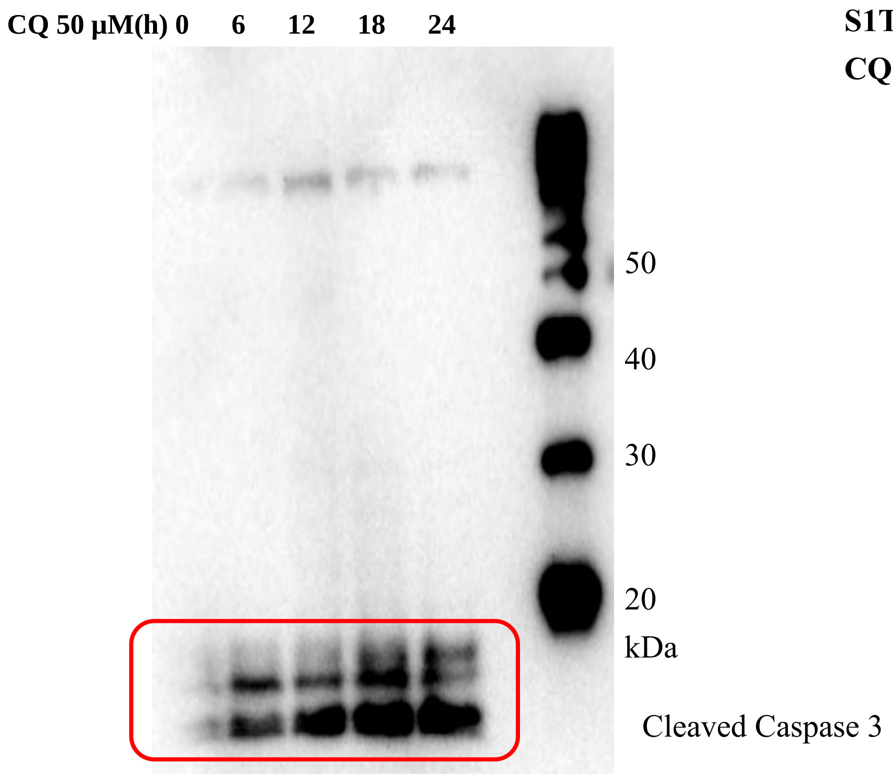

Fig 1C and Fig 2A

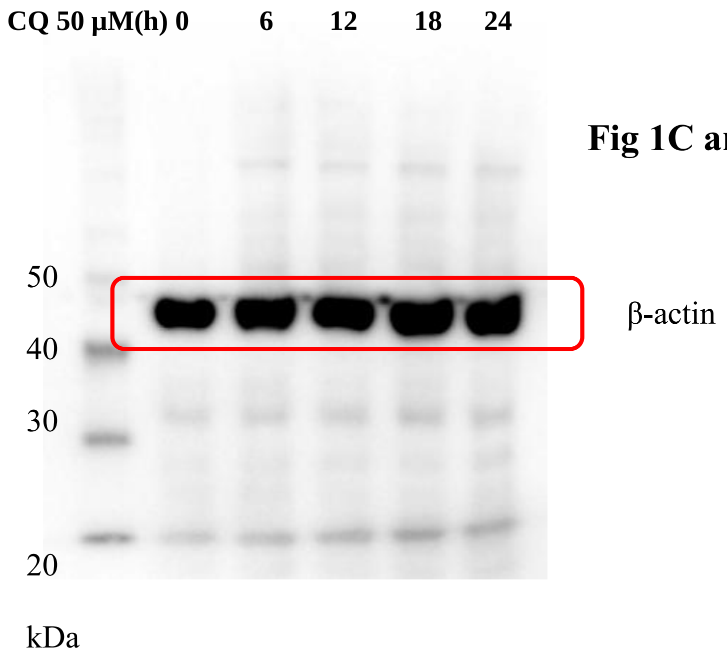

Fig 1C

S1T

HCQ

HCQ 25 μM(h) 0 6 12 18 24

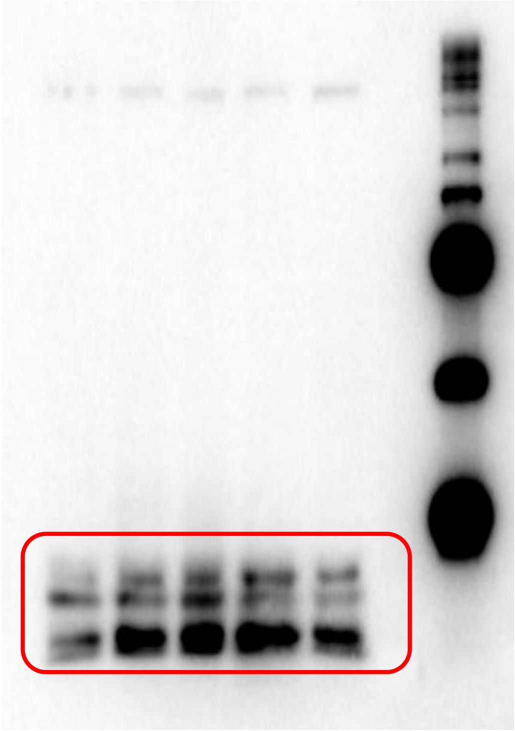

50
40
30
20
kDa

Cleaved Caspase 3

HCQ 25 μM(h) 0 6 12 18 24

Fig 1C and Fig 2A

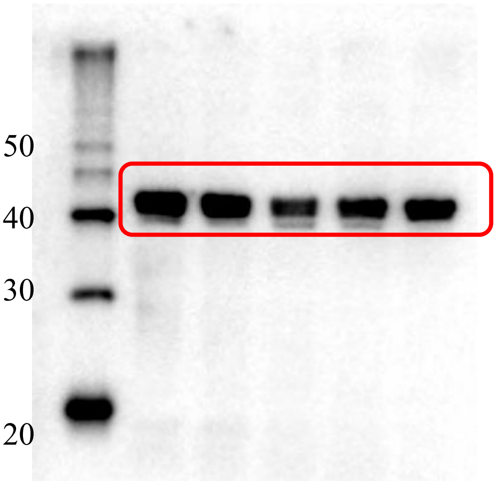

50
40
30
20
kDa

β-actin

Fig 1C

KK1

CQ

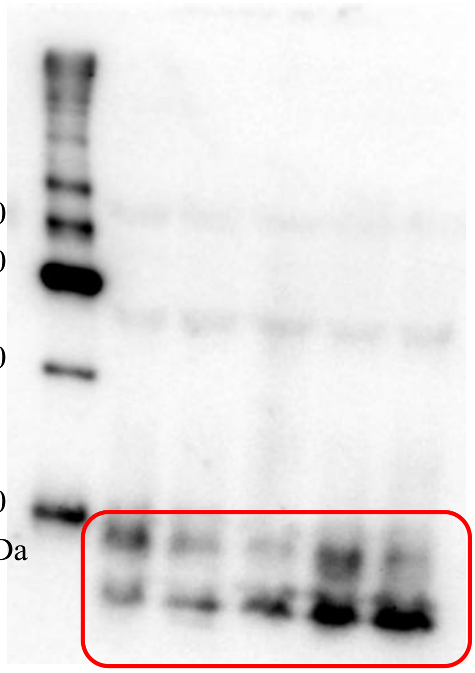

Fig 1C
and Supplementary Fig 2B

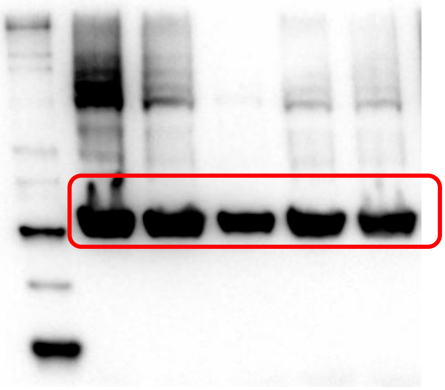

Fig 1C

KK1

HCQ

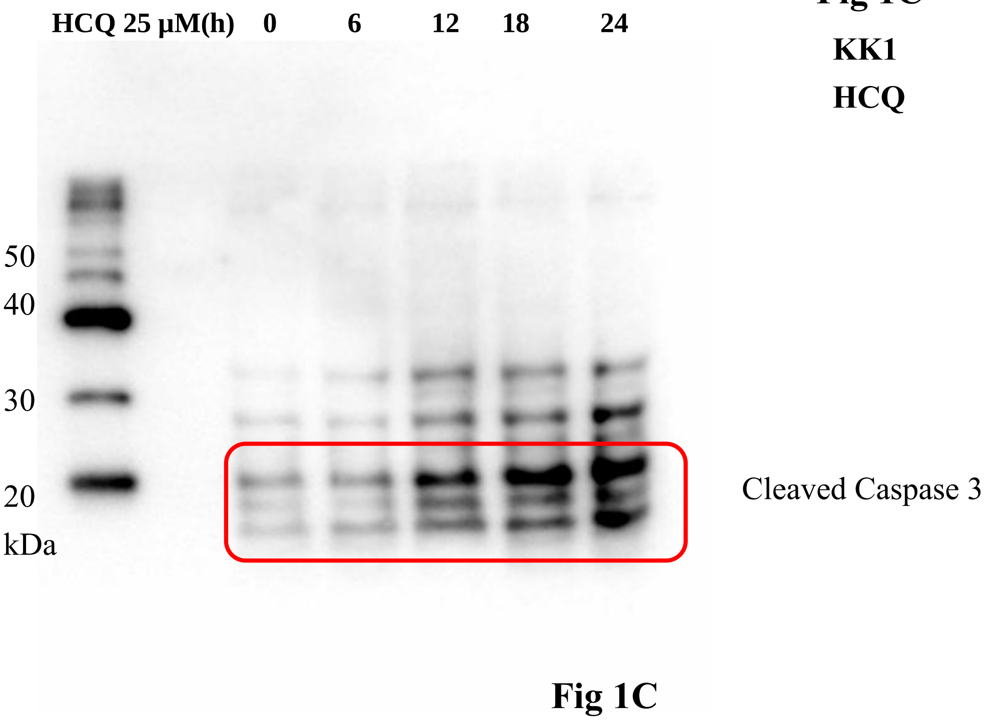

Fig 1C
and Supplementary Fig 2B

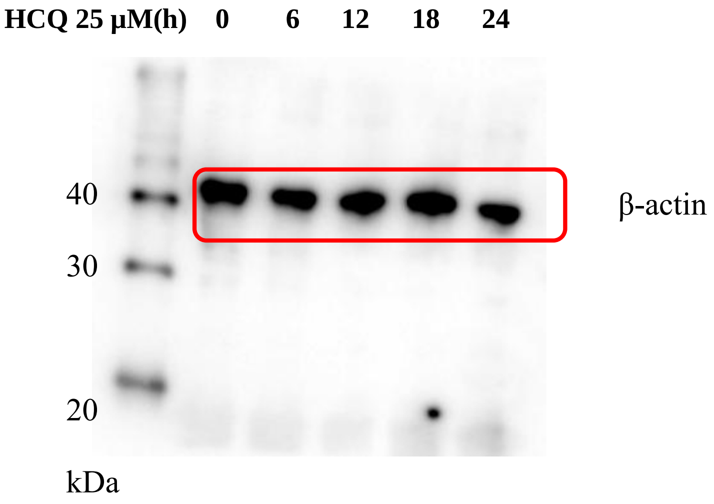

Fig 2A

S1T

CQ

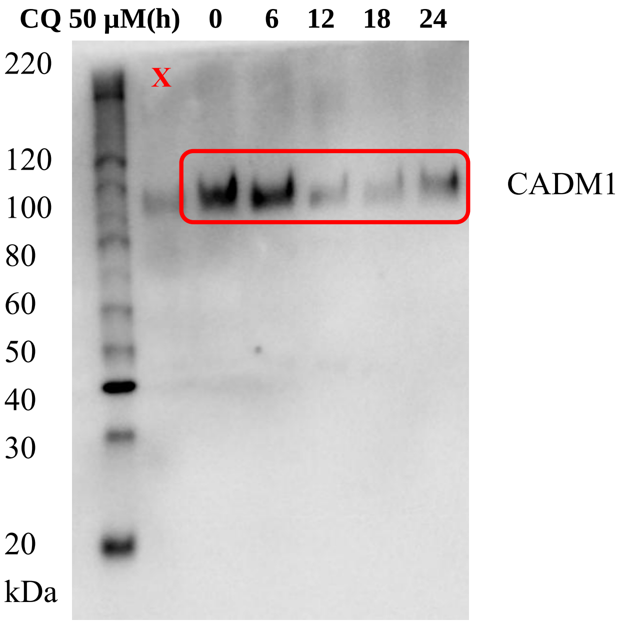
CQ 50 μM(h) 0 6 12 18 24
220
120
100
80
60
50
40
30
20
kDa
X
CADM1

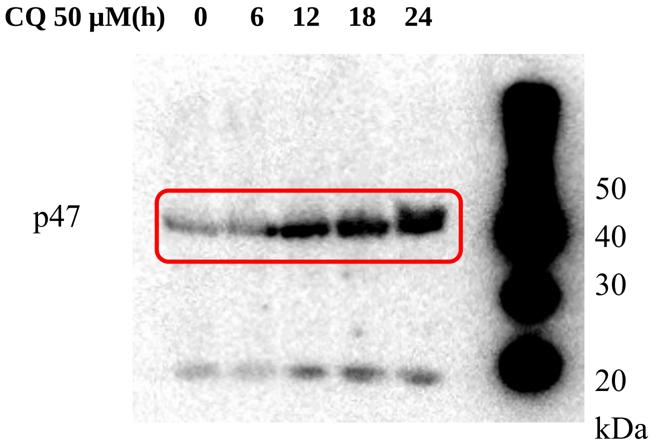
CQ 50 μM(h) 0 6 12 18 24
p47
50
40
30
20
kDa

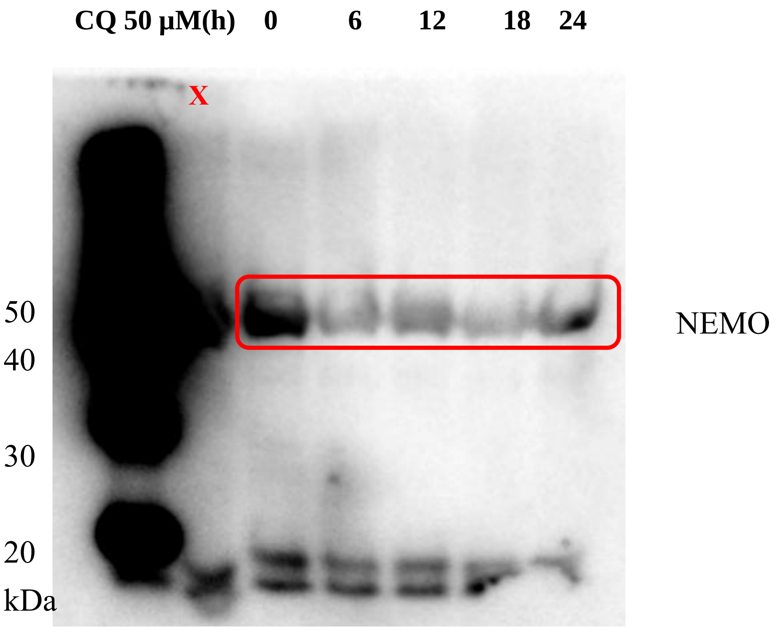
CQ 50 µM(h) 0 6 12 18 24
X
50
40
30
20
kDa
NEMO

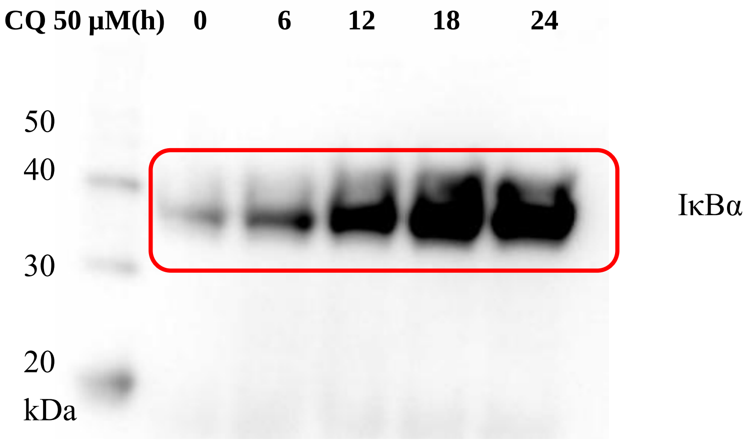
CQ 50 µM(h) 0 6 12 18 24
50
40
30
20
kDa
IκBα

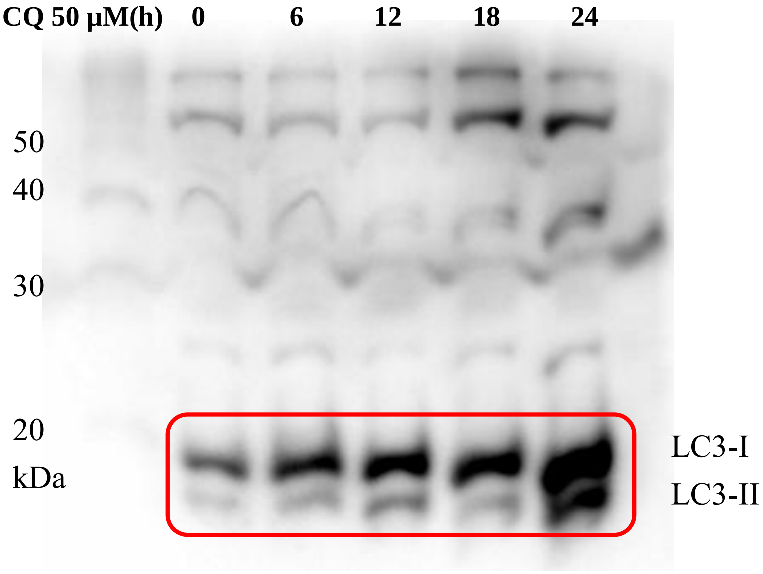
CQ 50 µM(h) 0 6 12 18 24
50
40
30
20
kDa
LC3-I
LC3-II

S1T

HCQ

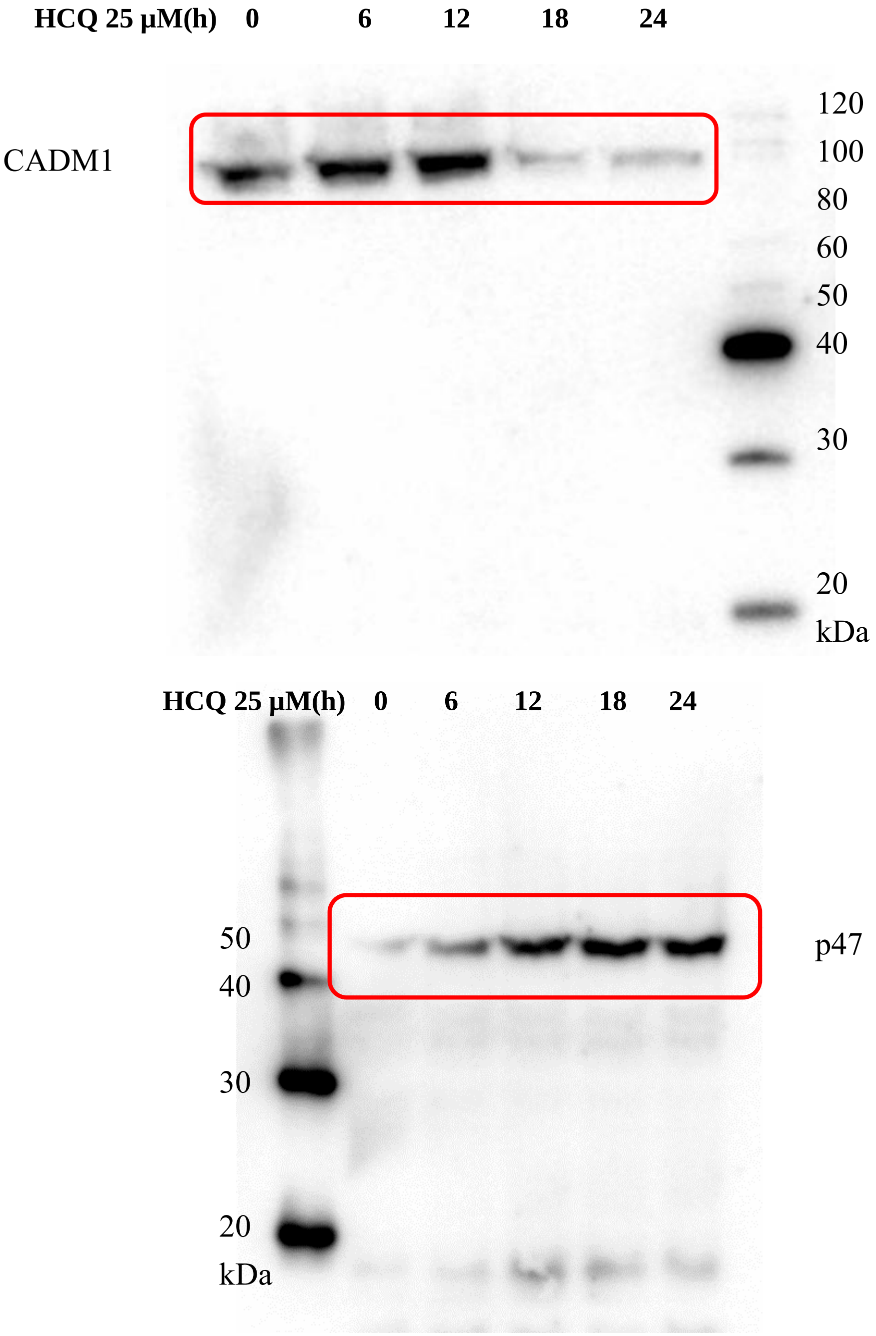
HCQ 25 μM(h) 0 6 12 18 24
CADM1
120
100
80
60
50
40
30
20
kDa
HCQ 25 μM(h) 0 6 12 18 24
50
40
30
20
kDa
p47

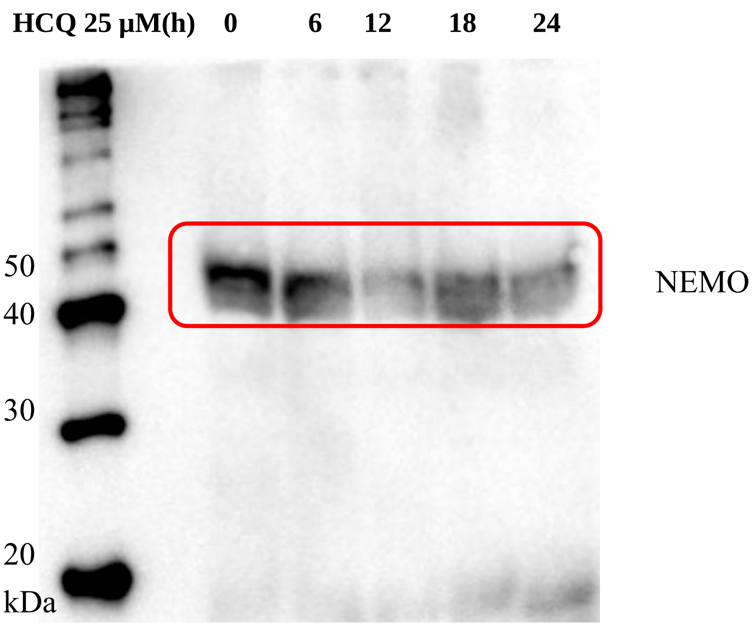
HCQ 25 μM(h) 0 6 12 18 24
50
40
30
20
kDa
NEMO

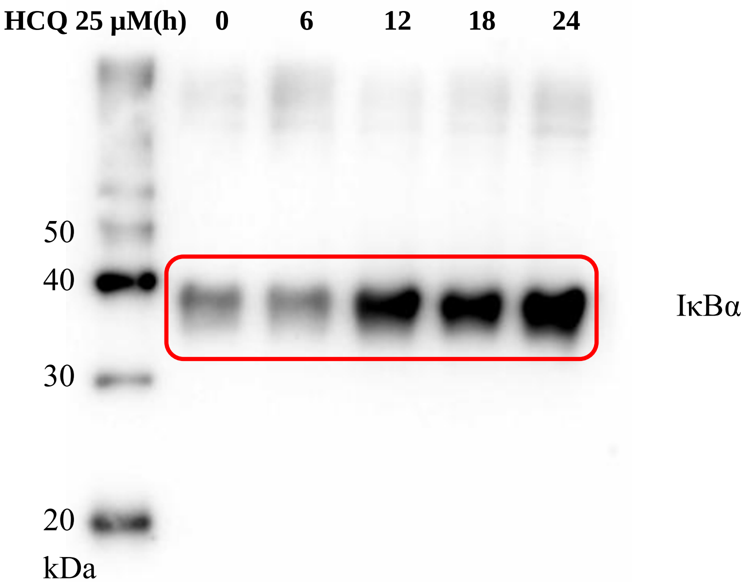
HCQ 25 μM(h) 0 6 12 18 24
50
40
30
20
kDa
IκBα

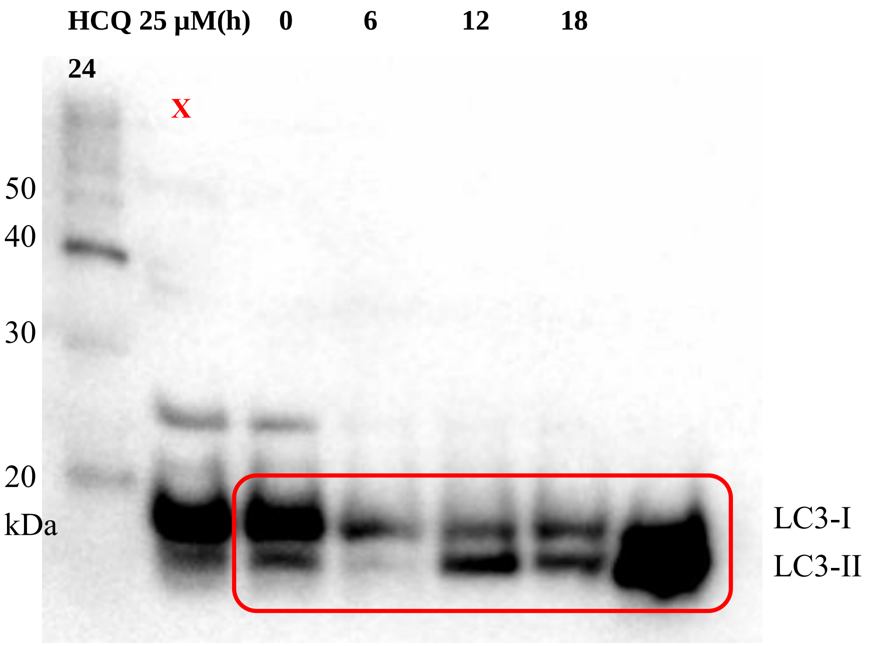
HCQ 25 μM(h) 0 6 12 18
24
X
50
40
30
20
kDa
LC3-I
LC3-II

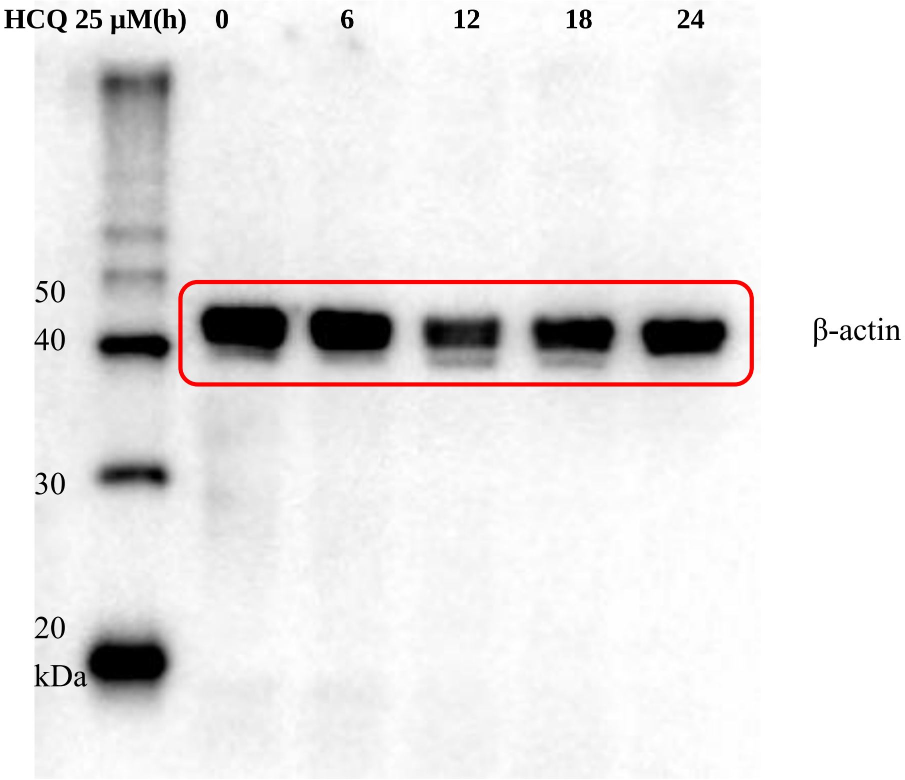
HCQ 25 μM(h) 0 6 12 18 24
50
40
30
20
kDa
β-actin

Supplementary Fig S2A

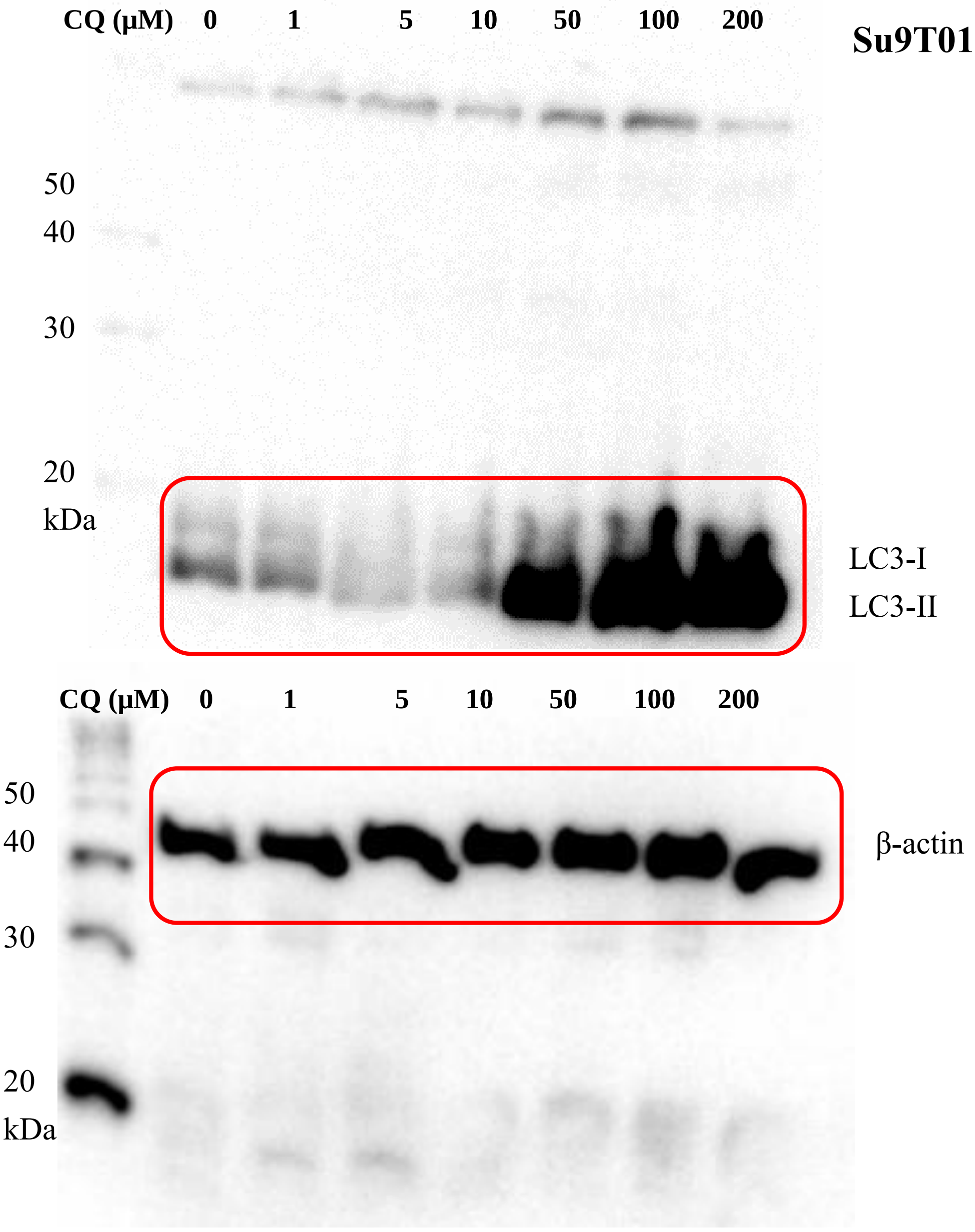

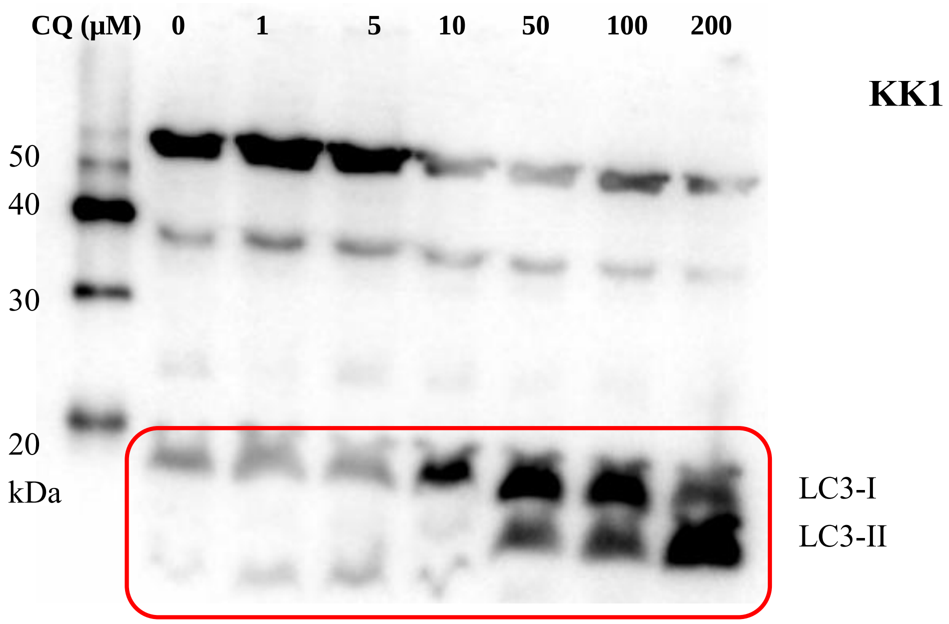
CQ (μM) 0 1 5 10 50 100 200
KK1
50
40
30
20
kDa
LC3-I
LC3-II

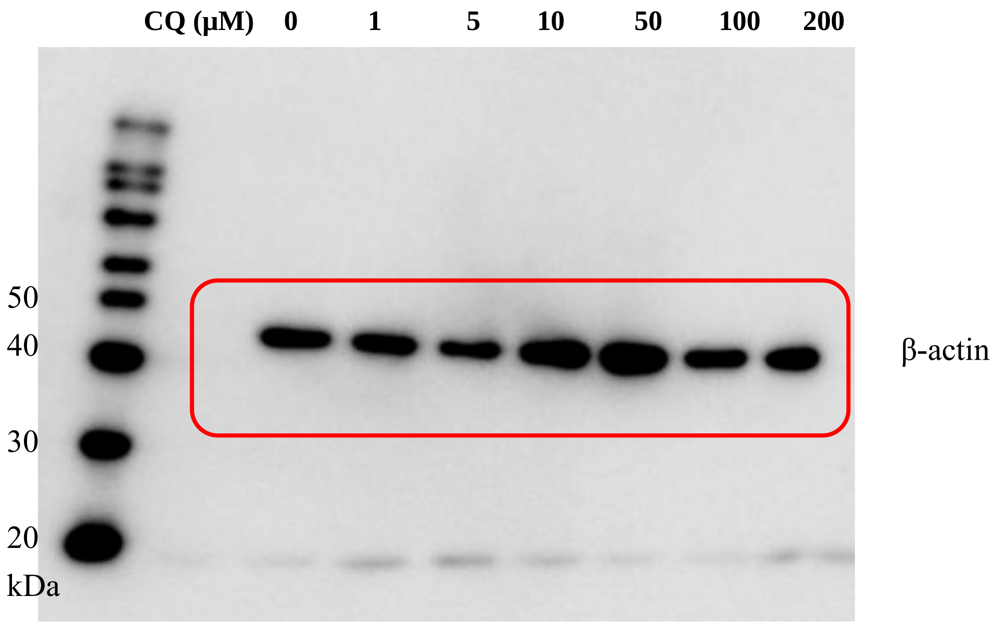
CQ (μM) 0 1 5 10 50 100 200
50
40
30
20
kDa
β-actin

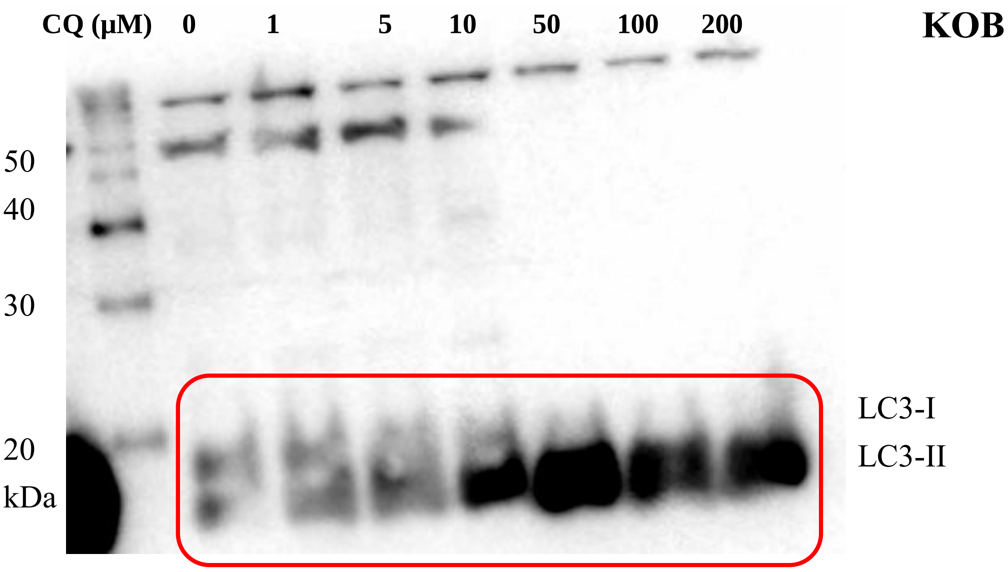
CQ (μM) 0 1 5 10 50 100 200
KOB
50
40
30
20
kDa
LC3-I
LC3-II

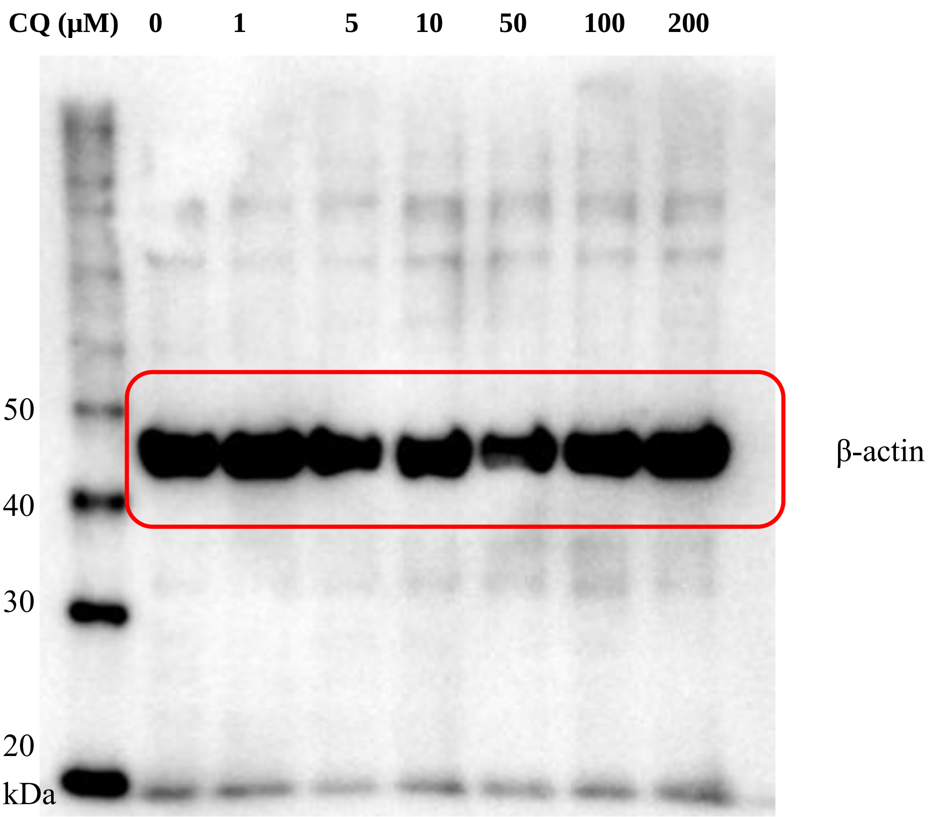
CQ (μM) 0 1 5 10 50 100 200
50
40
30
20
kDa
β-actin

S1T

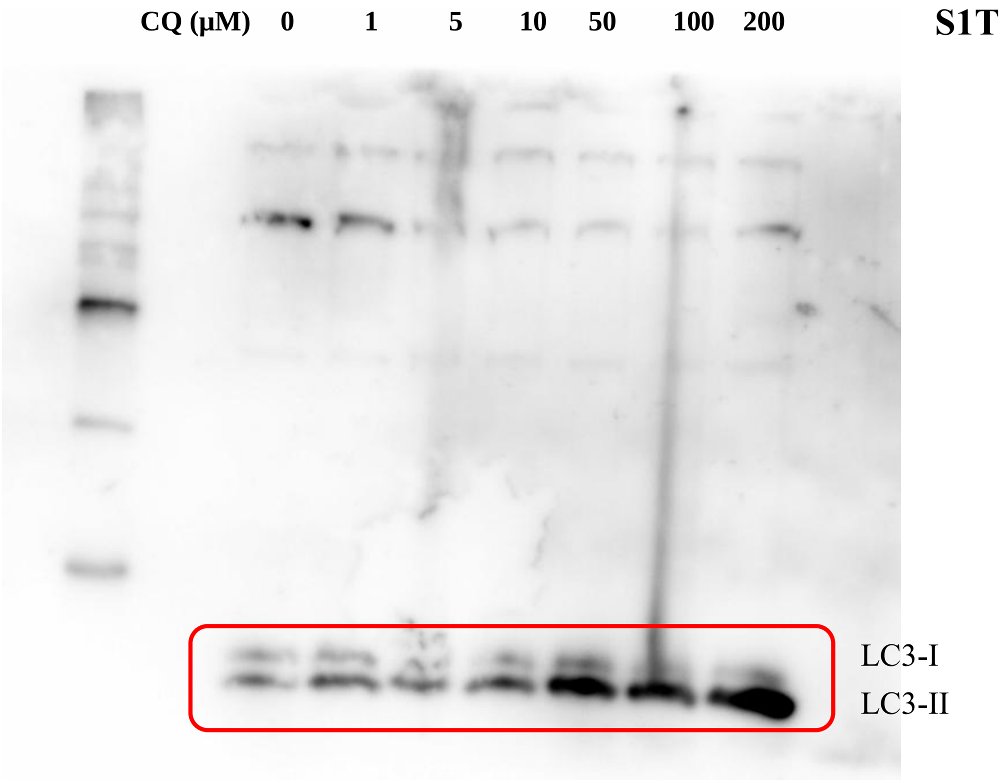

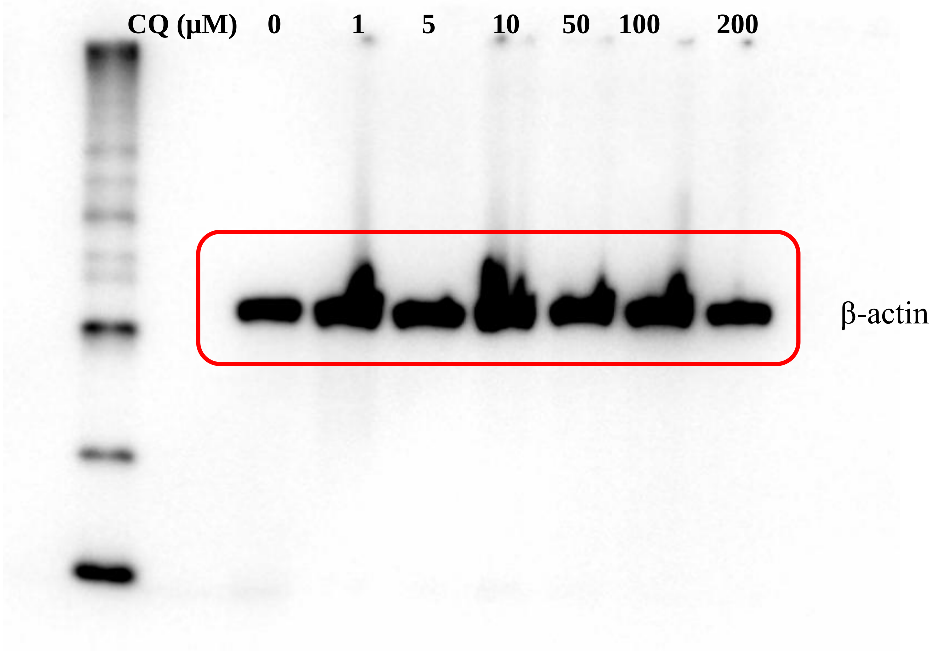

Supplementary Fig S2B

KK1
CQ

CQ 50 µM (h) **0** **6** **12** **18** **24**

CADM1

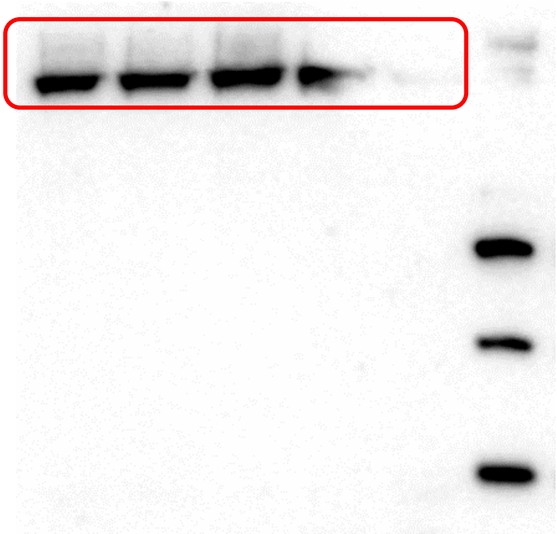

120
100
80
60
50
40
30
20
kDa

CQ 50 µM (h) **0** **6** **12** **18** **24**

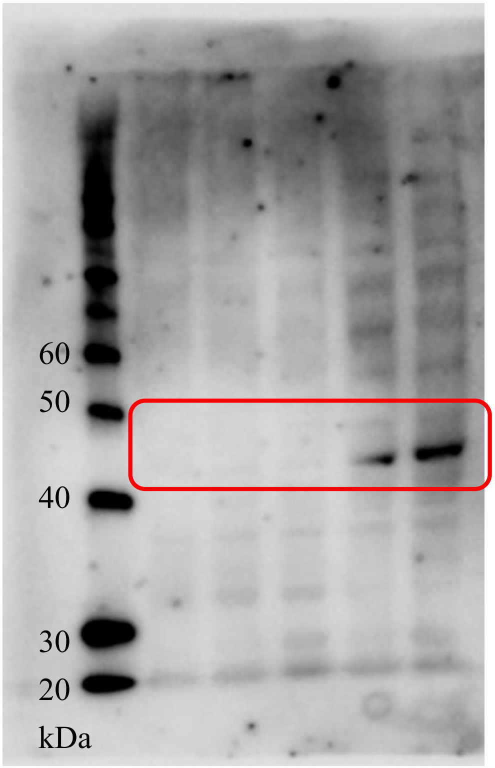

p47

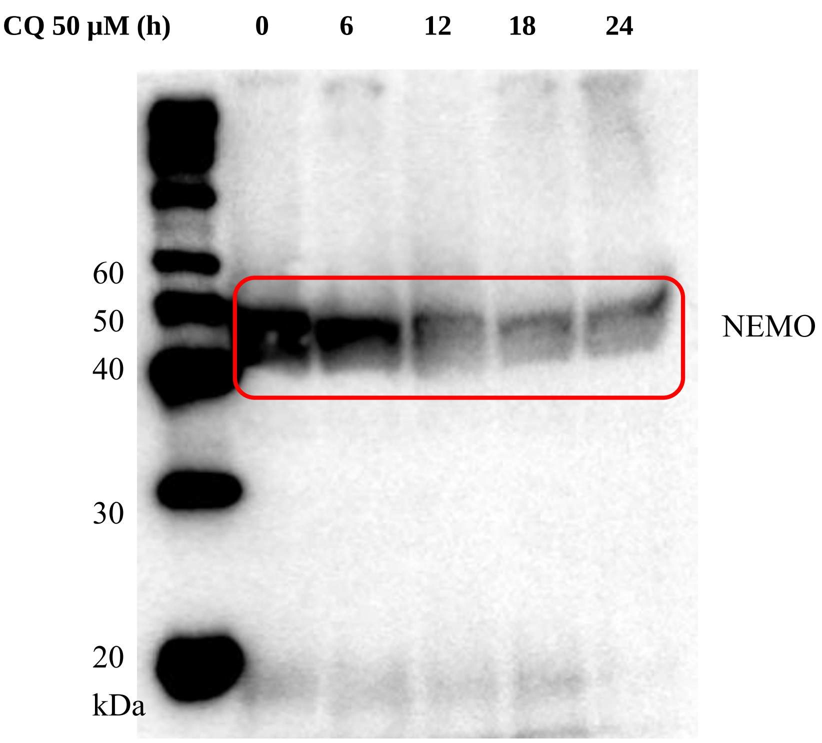
CQ 50 µM (h)
0
6
12
18
24
60
50
40
30
20
kDa
NEMO

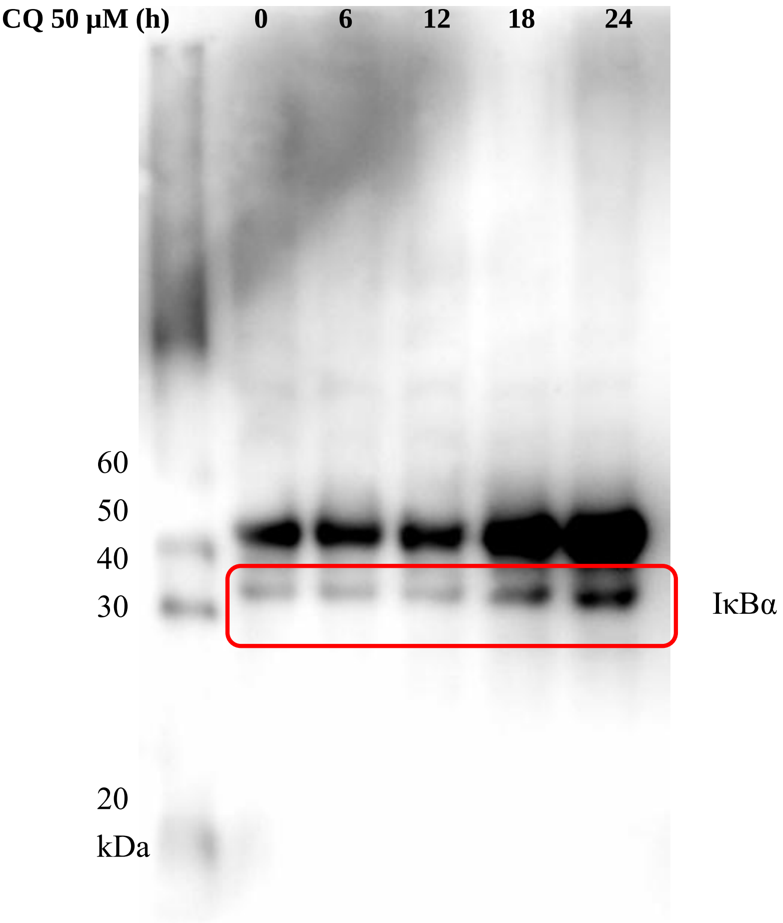
CQ 50 µM (h)
0
6
12
18
24
60
50
40
30
20
kDa
IκBα

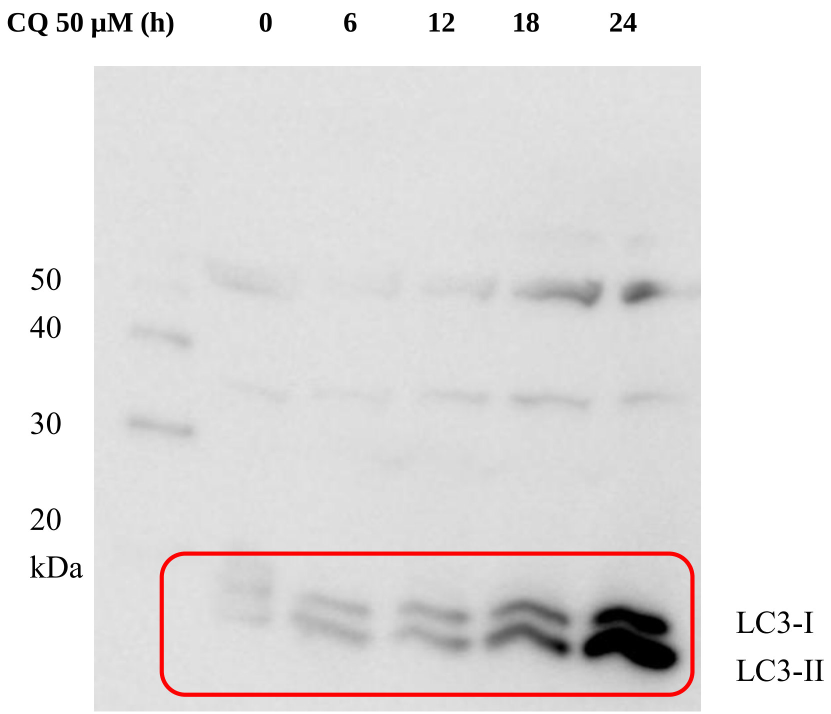
CQ 50 µM (h)
0
6
12
18
24
50
40
30
20
kDa
LC3-I
LC3-II

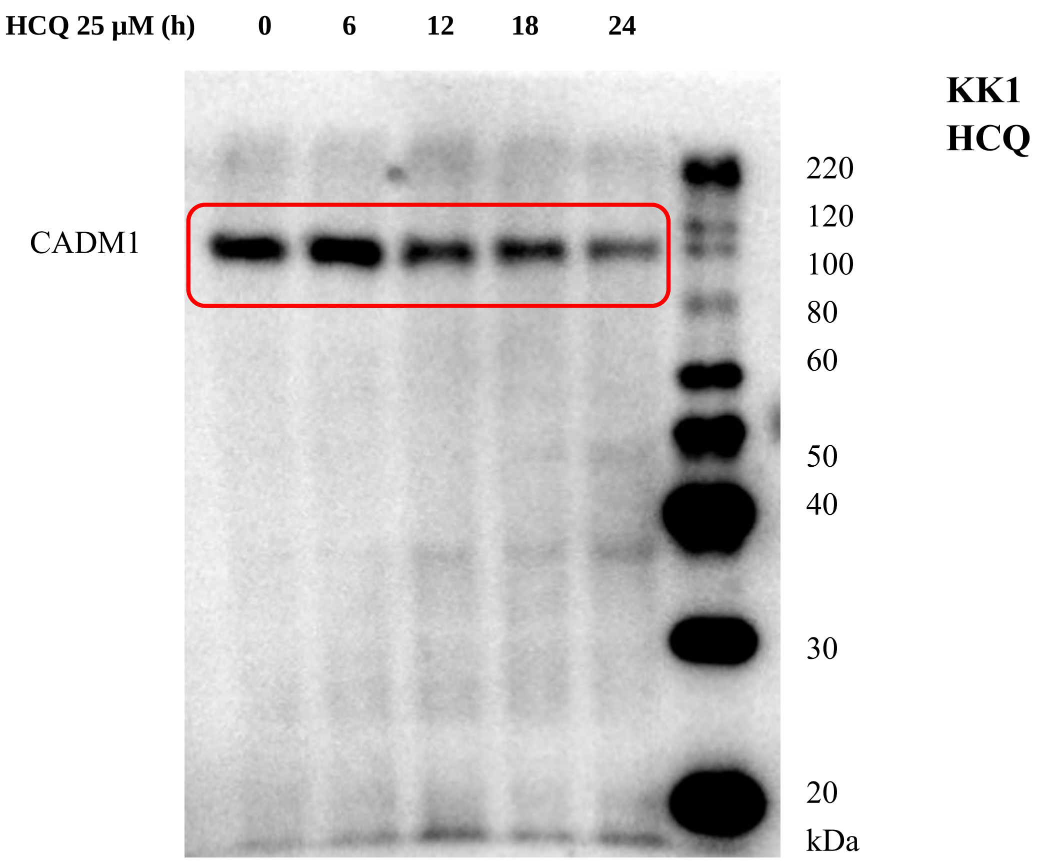
HCQ 25 µM (h)
0
6
12
18
24
KK1
HCQ
CADM1
220
120
100
80
60
50
40
30
20
kDa

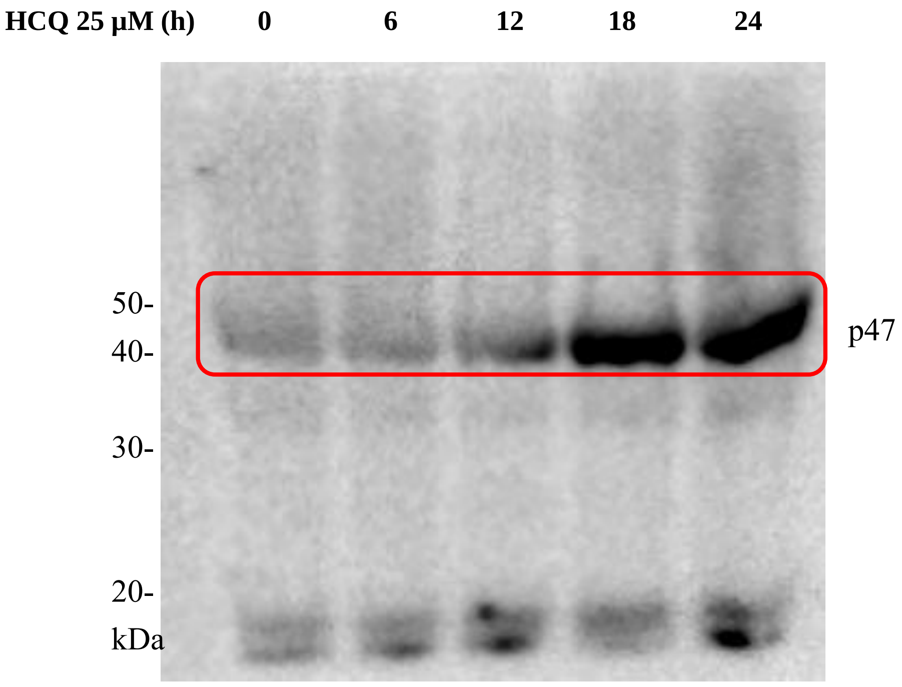
HCQ 25 µM (h)
0
6
12
18
24
50-
40-
30-
20-
kDa
p47

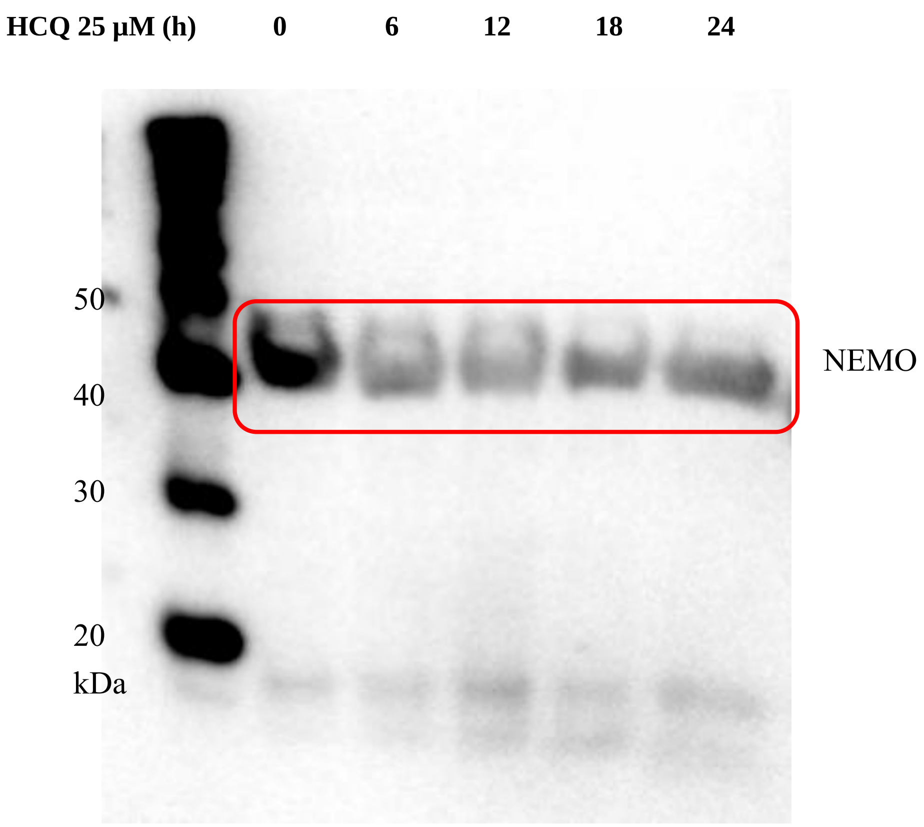
HCQ 25 μM (h)
0
6
12
18
24
50
40
30
20
kDa
NEMO

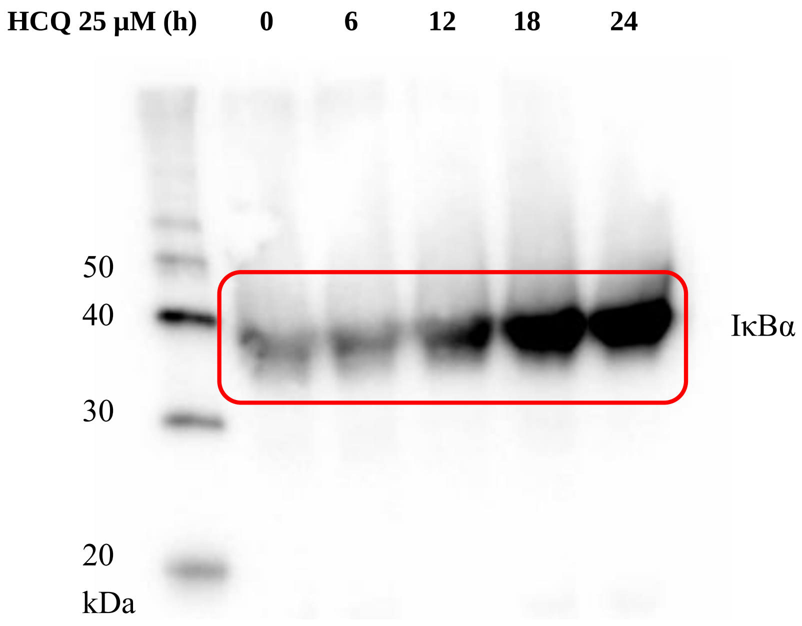
HCQ 25 μM (h)
0
6
12
18
24
50
40
30
20
kDa
IκBα

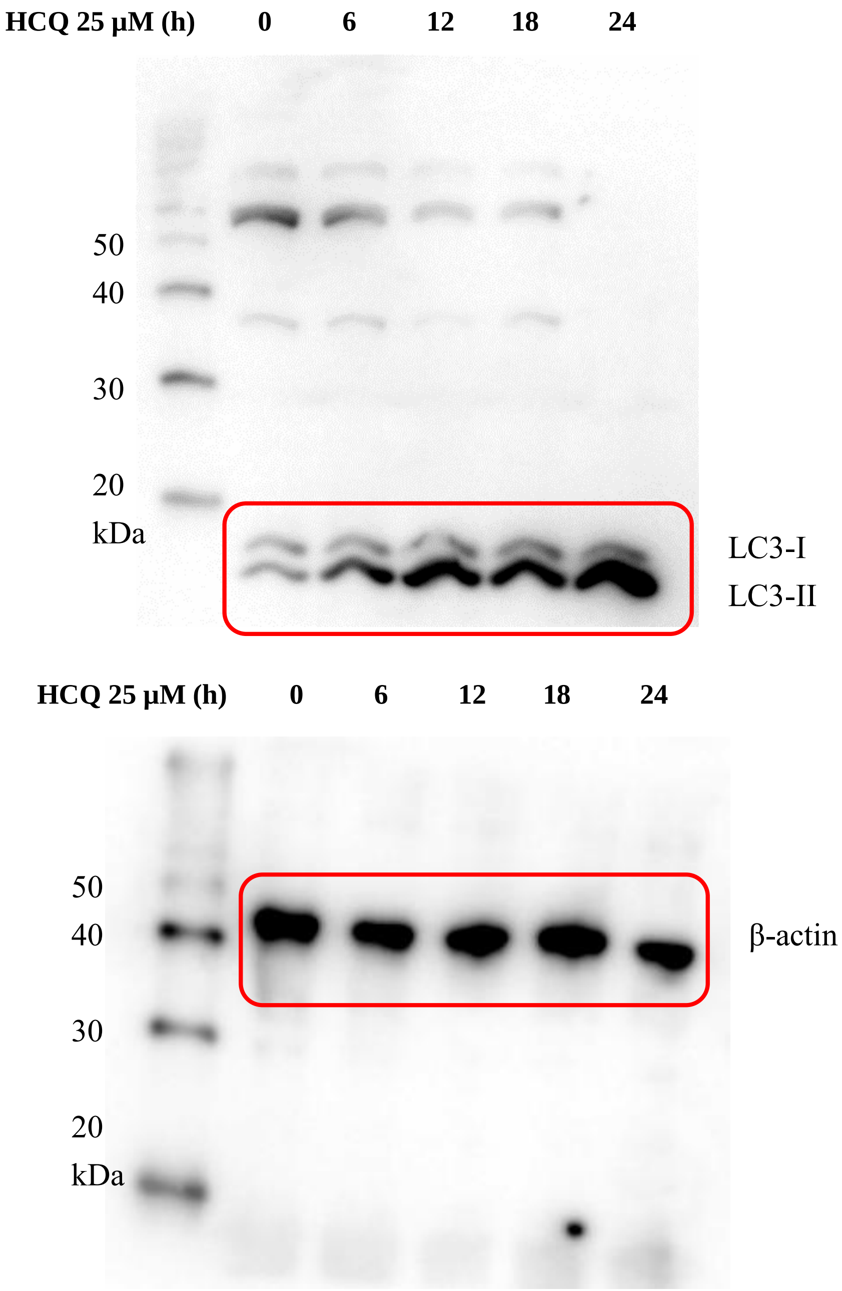
HCQ 25 µM (h)
0
6
12
18
24
50
40
30
20
kDa
LC3-I
LC3-II
HCQ 25 µM (h)
0
6
12
18
24
50
40
30
20
kDa
β-actin
