# Supplementary material for: Antitumor effects of chloroquine/hydroxychloroquine mediated by inhibition of the NF-κB signaling pathway through abrogation of autophagic p47 degradation in adult T-cell leukemia/lymphoma cells
Source: PLoS One. 2021 Aug 18;16(8):e0256320. doi: 10.1371/journal.pone.0256320 (PMC8372904; doi:10.1371/journal.pone.0256320)
Supplement: Attachment — Submitted filename: PONE-D-21-15148 REF revised.docx [file pone.0256320.s007.docx]

References

1. Poiesz BJ, Ruscetti FW, Gazdar AF, Bunn PA, Minna JD, Gallo RC. Detection and isolationof type C retrovirus particles from fresh and cultured lymphocytes of a patient with15cutaneous T-cell lymphoma. Proc Natl Acad Sci U S A. 1980;77: 7415–7419. doi:10.1073/pnas.77.12.7415

2. Gross C, Thoma-Kress AK. Molecular mechanisms of HTLV-1 cell-to-cell transmission. Viruses. 2016;8: 1–22. doi:10.3390/v8030074

3. ~~Fauquenoy S, Robette G, Kula A, Vanhulle C, Bouchat S, Delacourt N, et al. Repression of Human T-lymphotropic virus type 1 Long Terminal Repeat sense transcription by Sp1 recruitment to novel Sp1 binding sites. Sci Rep. 2017;7: 1–16. doi:10.1038/srep43221~~

[**Epidemiology**, **treatment**, and **prevention** of **human** **T**-**cell** **leukemia** virus type 1-associated diseases.](https://pubmed.ncbi.nlm.nih.gov/20610824/)

Gonçalves DU, Proietti FA, Ribas JG, Araújo MG, Pinheiro SR, Guedes AC, Carneiro-Proietti AB.Clin Microbiol Rev. 2010 Jul;23(3):577-89. doi: 10.1128/CMR.00063-09.PMID: 20610824

4. ~~Katsuya H, Islam S, Tan BJY, Ito J, Miyazato P, Matsuo M, et al. The Nature of the HTLV- 1 Provirus in Naturally Infected Individuals Analyzed by the Viral DNA-Capture-Seq Approach. Cell Rep. 2019;29: 724-735.e4. doi:10.1016/j.celrep.2019.09.016~~

[**Epidemiology** of **HTLV**-**1** Infection and ATL in Japan: An Update.](https://pubmed.ncbi.nlm.nih.gov/32547527/)

Iwanaga M.Front Microbiol. 2020 May 29;11:1124. doi: 10.3389/fmicb.2020.01124. eCollection 2020.PMID: 32547527

5. ~~Mortreux F, Gabet AS, Wattel E. Molecular and cellular aspects of HTLV-1 associated leukemogenesis in vivo. Leukemia. 2003;17: 26–38. doi:10.1038/sj.leu.2402777~~

[Regulation of Latency in the Human T Cell Leukemia Virus, HTLV-1.](https://pubmed.ncbi.nlm.nih.gov/31283437/)

**Bangham CRM**, Miura M, Kulkarni A, Matsuoka M.Annu Rev Virol. 2019 Sep 29;6(1):365-385. doi: 10.1146/annurev-virology-092818-015501. Epub 2019 Jul 5.PMID: 31283437

6. Matsuoka M. Human T-cell leukemia virus type I and adult T-cell leukemia. Oncogene. 2003;22: 5131–5140. doi:10.1038/sj.onc.1206551

7. ~~Jabbour M, Tuncer H, Castillo J, Butera J, Roy T, Pojani J, et al. Hematopoietic SCT for adult T-cell leukemia/lymphoma: A review. Bone Marrow Transplant. 2011;46: 1039–1044. doi:10.1038/bmt.2011.27~~

[Progress in Allogeneic Hematopoietic Cell Transplantation in Adult T-Cell Leukemia-Lymphoma.](https://pubmed.ncbi.nlm.nih.gov/31681185/)

**Utsunomiya A.**Front Microbiol. 2019 Oct 1;10:2235. doi: 10.3389/fmicb.2019.02235. eCollection 2019.PMID: 31681185

8. ~~Artesi M, Marçais A, Durkin K, Rosewick N, Hahaut V, Suarez F, et al. Monitoring molecular response in adult T-cell leukemia by high-throughput sequencing analysis of HTLV-1 clonality. Leukemia. 2017;31: 2532–2535. doi:10.1038/leu.2017.260~~

[Diagnostic Approaches and Established Treatments for Adult **T** Cell Leukemia Lymphoma.](https://pubmed.ncbi.nlm.nih.gov/32636814/)

Tsukasaki K, Marçais A, Nasr R, Kato K, Fukuda T, Hermine O, Bazarbachi A.Front Microbiol. 2020 Jun 19;11:1207. doi: 10.3389/fmicb.2020.01207. eCollection 2020.PMID: 32636814

9. ~~El Hajj H, Tsukasaki K, Cheminant M, Bazarbachi A, Watanabe T, Hermine O. Novel 16 Treatments of Adult T Cell Leukemia Lymphoma. Front Microbiol. 2020;11: 1–12. doi:10.3389/fmicb.2020.01062~~

[CCR4 and its ligands: from bench to bedside.](https://pubmed.ncbi.nlm.nih.gov/25087232/)

Yoshie O, Matsushima K.Int Immunol. 2015 Jan;27(1):11-20. doi: 10.1093/intimm/dxu079. Epub 2014 Aug 2.PMID: 25087232

10. Nakahata S, Saito Y, Marutsuka K, Hidaka T, Maeda K, Hatakeyama K, et al. Clinical significance of CADM1/TSLC1/IgSF4 expression in adult T-cell leukemia/lymphoma. Leukemia. 2012;26: 1238–1246. doi:10.1038/leu.2011.379

11. Dewan MZ, Takamatsu N, Hidaka T, Hatakeyama K, Nakahata S, Fujisawa J, et al. Critical Role for TSLC1 Expression in the Growth and Organ Infiltration of Adult T-Cell Leukemia Cells In Vivo. J Virol. 2008;82: 11958–11963. doi:10.1128/jvi.01149-08

12. ~~Kuramochi M, Fukuhara H, Nobukuni T, Kanbe T, Maruyama T, Ghosh HP, et al. TSLC1 is a tumor-suppressor gene in human non-small-cell lung cancer. Nat Genet. 2001;27: 427– 430. doi:10.1038/86934~~

[Overexpression of a cell adhesion molecule, **TSLC1**, as a possible molecular marker for acute-type adult T-cell leukemia.](https://pubmed.ncbi.nlm.nih.gov/15471956/)

Sasaki H, Nishikata I, Shiraga T, Akamatsu E, Fukami T, Hidaka T, Kubuki Y, Okayama A, Hamada K, Okabe H, Murakami Y, Tsubouchi H, Morishita K.Blood. 2005 Feb 1;105(3):1204-13. doi: 10.1182/blood-2004-03-1222. Epub 2004 Oct 7.PMID: 15471956

13. Sarkar B, Nishikata I, Nakahata S, Ichikawa T, Shiraga T, Saha HR, et al. Degradation of p47 by autophagy contributes to CADM1 overexpression in ATLL cells through the activation of NF-κB. Sci Rep. 2019;9: 1–14. doi:10.1038/s41598-019-39424-7

14. Liu T, Zhang L, Joo D, Sun SC. NF-κB signaling in inflammation. Signal Transduct Target Ther. 2017;2. doi:10.1038/sigtrans.2017.23

15. Sun SC, Yamaoka S. Activation of NF-κB by HTLV-I and implications for cell transformation. Oncogene. 2005;24: 5952–5964. doi:10.1038/sj.onc.1208969

16. Fochi S, Mutascio S, Bertazzoni U, Zipeto D, Romanelli MG. HTLV deregulation of the NF-κB pathway: An update on tax and antisense proteins role. Front Microbiol. 2018;9: 1– 8. doi:10.3389/fmicb.2018.00285 17
