# Supplementary material for: Antitumor effects of chloroquine/hydroxychloroquine mediated by inhibition of the NF-κB signaling pathway through abrogation of autophagic p47 degradation in adult T-cell leukemia/lymphoma cells
Source: PLoS One. 2021 Aug 18;16(8):e0256320. doi: 10.1371/journal.pone.0256320 (PMC8372904; doi:10.1371/journal.pone.0256320)
Supplement: Attachment — Submitted filename: Reviewers comments Letter.docx [file pone.0256320.s008.docx]

Response to Reviewers:

June 24^th^, 2021

Dr. Guillermo Velasco

Academic Editor at PLOS ONE
Reference: Manuscript Number: PONE-D-21-15148

“Antitumor effects of chloroquine/hydroxychloroquine mediated by inhibition of the NF-κB signaling pathway through abrogation of autophagic p47 degradation in adult T-cell leukemia/lymphoma cells”

Dear Dr. Guillermo Velasco:

We have revised the manuscript according to the editorial requests. We believe that we have answered all points raised during the review process and hope that the revised manuscript is now acceptable for publication.

Yours sincerely,
Kazuhiro Morishita MD., PhD
Director, HTLV-1/ATL Research, Education and Medical Facility, Faculty of Medicine, University of Miyazaki

Professor, Project for Advanced Medical Research and Development,

Project Research Division, Frontier Science Research Center, University of Miyazaki,

5200 Kihara, Kiyotake, Miyazaki, Miyazaki, JAPAN 889-1692

Phone: +81-985-85-9610 Fax: +81-985-85-0609
E-mail: [kmorishi@med.miyazaki-u.ac.jp](mailto:kmorishi@med.miyazaki-u.ac.jp)

Editor’s notes:

Journal Requirements:

When submitting your revision, we need you to address these additional requirements.

1. Please ensure that your manuscript meets PLOS ONE's style requirements, including those for file naming. The PLOS ONE style templates can be found at

<https://journals.plos.org/plosone/s/file?id=wjVg/PLOSOne_formatting_sample_main_body.pdf> and

<https://journals.plos.org/plosone/s/file?id=ba62/PLOSOne_formatting_sample_title_authors_affiliations.pdf>

In response to the editor’s comment, we have changed the writing style to follow PLOS ONE's style requirements. Please kindly let us know if we have missed something.

2. Thank you for stating the following in the Acknowledgments Section of your manuscript:

"The authors are grateful to Dr. Yasuaki Yamada (Nagasaki University, Japan) and Dr. Naomichi Arima (Kagoshima University, Japan) for providing cell lines. This work was supported in part by Grants-in-Aid for Scientific Research (B) (25293081 and 17H03581) (KM) from the Japan Society for the Promotion of Science (JSPS) and by the Takeda Science Foundation (KM)."

We note that you have provided funding information that is not currently declared in your Funding Statement. However, funding information should not appear in the Acknowledgments section or other areas of your manuscript. We will only publish funding information present in the Funding Statement section of the online submission form.

Please remove any funding-related text from the manuscript and let us know how you would like to update your Funding Statement. Currently, your Funding Statement reads as follows:

"This work was supported in part by Grants-in-Aid for Scientific Research (B) (25293081 and 17H03581) (KM) from the Japan Society for the Promotion of Science (JSPS) and by the Takeda Science Foundation (KM)."

Please include your amended statements within your cover letter; we will change the online submission form on your behalf.

In response to the editor’s comment, we have corrected the sentences, “"The authors are grateful to Dr. Yasuaki Yamada (Nagasaki University, Japan) and Dr. Naomichi Arima (Kagoshima University, Japan) for providing cell lines” in the Acknowledge section (page 16)

3. We note that you have stated that you will provide repository information for your data at acceptance. Should your manuscript be accepted for publication, we will hold it until you provide the relevant accession numbers or DOIs necessary to access your data. If you wish to make changes to your Data Availability statement, please describe these changes in your cover letter and we will update your Data Availability statement to reflect the information you provide.

In response to the editor’s comment, **we will provide the repository information.**

4. PLOS ONE now requires that authors provide the original uncropped and unadjusted images underlying all blot or gel results reported in a submission’s figures or Supporting Information files. This policy and the journal’s other requirements for blot/gel reporting and figure preparation are described in detail at https://journals.plos.org/plosone/s/figures#loc-blot-and-gel-reporting-requirements and https://journals.plos.org/plosone/s/figures#loc-preparing-figures-from-image-files. When you submit your revised manuscript, please ensure that your figures adhere fully to these guidelines and provide the original underlying images for all blot or gel data reported in your submission. See the following link for instructions on providing the original image data: https://journals.plos.org/plosone/s/figures#loc-original-images-for-blots-and-gels.

In your cover letter, please note whether your blot/gel image data are in Supporting Information or posted at a public data repository, provide the repository URL if relevant, and provide specific details as to which raw blot/gel images, if any, are not available. Email us at plosone@plos.org if you have any questions.

In response to the editor’s comment, we have created a separate file, named S1_raw_images.pdf

5. PLOS requires an ORCID iD for the corresponding author in Editorial Manager on papers submitted after December 6th, 2016. Please ensure that you have an ORCID iD and that it is validated in Editorial Manager. To do this, go to ‘Update my Information’ (in the upper left-hand corner of the main menu), and click on the Fetch/Validate link next to the ORCID field. This will take you to the ORCID site and allow you to create a new iD or authenticate a pre-existing iD in Editorial Manager. Please see the following video for instructions on linking an ORCID iD to your Editorial Manager account: <https://www.youtube.com/watch?v=_xcclfuvtxQ>

In response to the editor’s comment, **we have authenticated the corresponding author’s ORCID iD**

6. Please cite Supporting Information Figure S5 in your main manuscript.

In response to the reviewer’s comment, we moved Figure S5 to Figure 4 and added paragraph to the Discussion section

Reviewer #1:

1. The authors should replace most of the references to the proper ones in the introduction section. I attached revised ref lists for their conveniences.

In response to the reviewer’s comment, we have replaced the suggested references (References number 3, 4, 5, 7, 8, 9, and 12).

2. No description of the PBL used in Fig.1A&B. in the Methods section. They should describe how they collected from healthy volunteers.

In response to the reviewer’s comment, we added the sentences “Informed consent was obtained from all the patients and the healthy donor” in the Methods-Patient samples section (page 7).

3. In clinical use, HCQ is less toxic than CQ. In Fig1A&B however, PBL viability seems dropping with less amount of HCQ than CQ. Please rationalize this discordance.

Our study showed that IC50 of HCQ was lower than CQ. Consistent results with other cells (ATLL, T-ALL and HAM-TSP cells), indicating that HCQ is more potent than CQ. So, to reach a similar effect, HCQ needs a lower dose than CQ. Regarding toxicity, our in vivo studies using a recommended maintenance dose of HCQ for SLE in humans, and we did not observe any particular side effects during experiments.

4. Cmax of CQ is around 500nM. The viability of PBL and ATL cells look quite similar at this concentration. How the authors improve the efficacy of CQ/HCQ in clinical practice? It seems difficult to have sufficient efficacy with single drug treatment.

We understand that at 500nM our in vitro data does not show the desired effect. However, our in vivo study used a clinical maintenance recommended dose for SLE, and it showed a significant tumor growth inhibition and extended the survival. Currently, we do not have any data to explain this in vitro-in vivo discrepancy.

Reviewer #2: The study presented by Yanuar Rahmat Fauzi et al. entitled “Antitumor effects of chloroquine/hydroxychloroquine mediated by inhibition of the NF-κB signaling pathway through abrogation of autophagic p47 degradation in adult T-cell leukemia/lymphoma cells” shows that CQ and HCQ inhibit ATLL cell growth in in vitro assays as well as in a mouse model for ATLL. The autophagy inhibition mediated by CQ or HCQ promoted p47 protein recovery and inhibition of NF-κB activation, leading to apoptosis in ATLL cell lines.

The manuscript presents interesting data, it is novel, and conclusions are supported by the experimental data. However, the issues listed below should be addressed.

Major Points:

1. Page 9. Authors claim that CQ and HCQ impair ATLL cell growth. However, they perform a MTT assay that assays metabolically active cells, then MTT is an indirect parameter of viability instead of cell growth. There are many situations where cells might not divide and be metabolically active. A measurement with trypan blue, or a supravital stain is recommended.

In response to the reviewer’s comment, we performed a growth assay measured by trypan blue. The results showed that there was significance difference cell growth between KK1, S1T, MT2, MOLT4 and JURKAT compared to healthy PBMC (Fig S1 B). We added citation, sentences and figures in the Result and Supplementary methods section (page 9, Figure S1B)

2. Figure 2. Treatment with different CQ concentration and LC3 western blot for ST1 cell line should be depicted.

We think the reviewer mistyping S1T as ST1, as the correlated cell line in the figure 2 is S1T.

In response to the reviewer’s comment, we assayed the LC3 turnover of S1T that were either untreated or treated with various doses of CQ. The results showed increased levels of LC3-II in a dose dependent pattern (Fig S2A). We added sentences and figures in the Result section (page 11, Figure S1B)

3. The authors state that CQ treatment causes accumulation of autophagosomes. They support that state by the increased level of LC3-II in all ATLL cell lines in WB (figure S2A). It would be nice to show the increased LC3 positive dots by immunofluorescence.

In response to the reviewer’s comment, we performed an immunofluorescence assay and quantified the puncta. The results showed that CQ/HCQ treatment significantly increased the LC3 expression. We added sentences and figures in the Result section (page 11, Figure 2C and S2D)

4. Page 11. Text says that the number of apoptotic cells in tumor xenografts was significantly increased by treatment with HCQ. However, authors do not show any statistical analysis to support such a thing. It would be necessary to quantify the caspase 3 positive cells or alternatively use another methodology such as TUNEL assay in xenografts and apply the appropriate statistical analysis.

In response to the reviewer’s comment, we quantified the caspase 3 stained cells and analyzed the data. The results showed that the number of caspase 3 stained cells in tumor xenografts was significantly increased by treatment with HCQ. We added the sentences and figures in the results section (page 12, Figure 3G)

5. The authors used a Su9To1 cell injection model in the tail vein of mice, then survival was monitored. The authors must clarify this model better in Methods section. Additionally, they should give an explanation that justify the use of this model and/or insert a citation where the model is described.

In response to the reviewer’s comment, we added sentences, “After 3 days, the mice were randomly divided into 2 groups (water or 60 mg/kg bw/day HCQ, p.o.), and treatment was started immediately. HCQ or a control vehicle (water) was orally given for a 16-day period”, and insert the citation (page 9)

Minor Points:

1. Clarify in the text that autophagy refers to macroautophagy.

As the reviewer noted, we added the sentences “An important mechanism of the activated NF-κB signaling pathway in ATLL is the activation of the macroautophagy(herafter referred to as autophagy in the remainder of this manuscript)-lysosomal degradation of p47 (NSFL1C), a negative regulator of the NF-κB pathway” in the Abstract section (Page 3).

2. In the title “CQ and HCQ inhibited autophagy and the canonical NF-κB pathway” it must be disclosed that it is in ATLL cells lines, so, it should be “CQ and HCQ inhibited autophagy and the canonical NF-κB pathway in ATLL cells lines”.

As the reviewer noted, we corrected the title “CQ and HCQ inhibited autophagy and the canonical NF-κB pathway” to “CQ and HCQ inhibited autophagy and the canonical NF-κB pathway in ATLL cells lines” in the Results section (page 10) .

3. Figure S2C, authors should clarify if LC3-II turnover calculation is from S2B WB.

As the reviewer noted, we changed the sentences “LC3-II turnover is presented as the mean ± SD (n = 2). *p < 0.05, **p<0.01 compared to the control” to “LC3-II turnover is presented as the mean ± SD (n = 2 independent experiments, representative blot was shown at Fig 2 A). *p < 0.05, **p<0.01 compared to the control” in the figure legend

4. To enhance an easy reading, the results from figure S1 B and C must be included in Figure 1.

As the reviewer noted, we moved the “Figure S1 B and C” to the “Figure 1 C and D.

5. How do authors explain the behavior of HTLV-1-infected T cell lines derived from HAM-TSP patients (HCT1, HCT4 and HCT5) in figure S1A?

As the reviewer noted, we edited the sentences “Interestingly, CQ or HCQ treatment also inhibited cell growth in HTLV-1-infected T cell lines derived from HTLV-1-associated myelopathy (HAM)/tropical spastic paraparesis (TSP) patients (**Fig S1A**)” to “Interestingly, CQ or HCQ treatment also inhibited cell growth in HTLV-1-infected T cell lines derived from HTLV-1-associated myelopathy (HAM)/tropical spastic paraparesis (TSP) patients in a dose-dependent manner (IC50 CQ = 34.73 +/- 24.89 and IC50 HCQ = 30.37 +/- 12.46) (**Fig S1A**)” in the Results section (page 9).

6. Figure S3. Please, clarify which cell line corresponds to each picture.

As the reviewer noted, we added the label to each group

(A. Su9T1 cell line xenografted mice, B. MT2 cell line xenografted mice and C. Su9T1 cell line xenografted mice)

7. In Figure 3, authors have to clarify images’ labels.

As the reviewer noted, we added the label to each group

(A. Su9T01 cell line xenografted mice, B. MT2 cell line xenografted mice and C. Su9T01 cell line xenografted mice)

8. In the title "CQ and HCQ inhibit tumor growth and extend the lifespan of ATLL cells in xenograft mice", Do CQ and HCQ extend the lifespan of ATLL cells or the mice?

As the reviewer noted, we corrected the title "CQ and HCQ inhibit tumor growth and extend the lifespan of ATLL cells in xenograft mice" to “CQ and HCQ inhibit tumor growth and extend the lifespan of ATLL cells xenografted mice” in the Results Section (page 11)

9. It would be nice to transfer to the discussion section the proposed model in Figure S5.

As the reviewer noted, we moved the proposed model in figure S5 to main figure 4, and added the paragraph “To sum up, we propose an anti-tumor model of CQ/HCQ in ATLL cells in Fig 4. CQ/HCQ treatment inhibited the high autophagic flux of ATLL cells. At this point, CQ/HCQ rescues p47 protein from the autophagy-lysosomal degradation pathway. The abundant p47 inhibited NEMO-IKK complex interaction, leading to downregulation of NEMO and preventing IKba degradation. Finally, the inhibition of NF-kB activation induced downregulation of CADM1 and caspase-related apoptosis” in the Discussion section (page 14)
