# Supplementary material for: Antitumor effects of chloroquine/hydroxychloroquine mediated by inhibition of the NF-κB signaling pathway through abrogation of autophagic p47 degradation in adult T-cell leukemia/lymphoma cells
Source: PLoS One. 2021 Aug 18;16(8):e0256320. doi: 10.1371/journal.pone.0256320 (PMC8372904; doi:10.1371/journal.pone.0256320)
Supplement: Attachment — Submitted filename: Response to Reviewers-20210722.docx [file pone.0256320.s009.docx]

Response to Reviewers:

July 22^nd^, 2021

Dr. Guillermo Velasco

Academic Editor at PLOS ONE
Reference: Manuscript Number: PONE-D-21-15148R1

“Antitumor effects of chloroquine/hydroxychloroquine mediated by inhibition of the NF-κB signaling pathway through abrogation of autophagic p47 degradation in adult T-cell leukemia/lymphoma cells”

Dear Dr. Guillermo Velasco:

We have revised the manuscript according to the editorial requests. We believe that we have answered all points raised during the review process and hope that the revised manuscript is now acceptable for publication.

Yours sincerely,
Kazuhiro Morishita MD., PhD
Director, HTLV-1/ATL Research, Education and Medical Facility, Faculty of Medicine, University of Miyazaki

Professor, Project for Advanced Medical Research and Development,

Project Research Division, Frontier Science Research Center, University of Miyazaki,

5200 Kihara, Kiyotake, Miyazaki, Miyazaki, JAPAN 889-1692

Phone: +81-985-85-9610 Fax: +81-985-85-0609
E-mail: [kmorishi@med.miyazaki-u.ac.jp](mailto:kmorishi@med.miyazaki-u.ac.jp)

Editor’s notes:

Journal Requirements:

Please include the following items when submitting your revised manuscript:

- A rebuttal letter that responds to each point raised by the academic editor and reviewer(s). You should upload this letter as a separate file labeled 'Response to Reviewers'.
- A marked-up copy of your manuscript that highlights changes made to the original version. You should upload this as a separate file labeled 'Revised Manuscript with Track Changes'.
- An unmarked version of your revised paper without tracked changes. You should upload this as a separate file labeled 'Manuscript'.

Reviewer #1: They nicely reorganized their first manuscript to the revised one including some additional data and texts.

Reviewer #2: The revised manuscript by Yanuar Rahmat Fauzi et al. has been improved and authors have responded to my questions and concerns. However, I still see necessary to clarify the following three points:

1. Please, explain in the materials and methods section, how LC3 positive dots were quantified in immunofluorescence (Figure S2D), and mainly the used criteria.

In response to the reviewer’s comment, we added the sentences “Average numbers of LC3 puncta/cell were quantified by blinded manual counting of puncta, with at least 30 cells counted per group.” in the Supplementary Methods section (page 6).

2. On the same line, with the previous point, please explain in the materials and methods section, quantification criteria for caspase 3 in Figure 3G.

In response to the reviewer’s comment, we added the sentences “Quantification of anti-cleaved caspase-3 positive cells was performed by blinded manual counting the number of positive cells/High Power Field (HPF).” in the Supplementary Methods section (page 7).

3. The quality of figures 3 D and E should be improved.

In response to the reviewer’s comment, we have changed figures 3 D and E.
